# First principle simulation of coated hydroxychloroquine on Ag, Au and Pt nanoparticles

**DOI:** 10.1038/s41598-021-81617-6

**Published:** 2021-01-22

**Authors:** Razieh Morad, Mahmood Akbari, Parham Rezaee, Amin Koochaki, Malik Maaza, Zahra Jamshidi

**Affiliations:** 1 UNESCO-UNISA Africa Chair in Nanoscience and Nanotechnology (U2ACN2), College of Graduate Studies, University of South Africa (UNISA), Pretoria, South Africa; 2 Material Research Division, Nanoscience African Network (NANOAFNET), iThemba LABS-National Research Foundation, Somerset West, 7129 South Africa; 3 Chemistry Department, Sharif University of Technology, 11155-9516 Tehran, Iran

**Keywords:** Biochemistry, Computational biology and bioinformatics, Molecular medicine, Nanoscience and technology

## Abstract

From the first month of the COVID-19 pandemic, the potential antiviral properties of hydroxychloroquine (HCQ) and chloroquine (CQ) against SARS-CoV-2 suggested that these drugs could be the appropriate therapeutic candidates. However, their side effects directed clinical tests towards optimizing safe utilization strategies. The noble metal nanoparticles (NP) are promising materials with antiviral and antibacterial properties that can deliver the drug to the target agent, thereby reducing the side effects. In this work, we applied both the quantum mechanical and classical atomistic molecular dynamics approaches to demonstrate the adsorption properties of HCQ/CQ on Ag, Au, AgAu, and Pt nanoparticles. We found the adsorption energies of HCQ/CQ towards nanoparticles have the following trend: PtNP > AuNP > AuAgNP > AgNP. This shows that PtNP has the highest affinity in comparison to the other types of nanoparticles. The (non)perturbative effects of this drug on the plasmonic absorption spectra of AgNP and AuNP with the time-dependent density functional theory. The effect of size and composition of NPs on the coating with HCQ and CQ were obtained to propose the appropriate candidate for drug delivery. This kind of modeling could help experimental groups to find efficient and safe therapies.

## Introduction

Given that the process of developing new drugs to become appropriate clinical candidates is extensive, one of the most rapid and reliable treatments is drug repurposing—the examination of existing FDA approved drugs for new therapeutic purposes^1^. Chloroquine (CQ) and hydroxychloroquine (HCQ) have been used for many years as pharmacotherapies for malaria and were recently proposed as a potential therapeutic option against COVID-19^2^. The pre-clinical studies have shown the prophylactic and antiviral effects of CQ and HCQ against SARS-CoV-2 (or COVID-19)^2–6^. The clinical safety profile for HCQ is better than that of CQ, thus allowing for long-term usage and higher daily dosage^7^. Some reports have mentioned that large scale (and prolonged) usage is potentially harmful and increases the risk of drug-induced torsades de pointes and may lead to cardiac death^8–10^. Therefore, different treatment regimens try to focus on efficient strategies for in vivo usage of these drugs^11,12^, especially the balance between the concentration of the drug in the blood and its severe potential toxicity, to ensure the safety of these therapeutic strategies^7,13^. Despite conflicting evidence on the efficiency of HCQ for the treatment of COVID-19, the recent clinical studies have reported no potent evidence to support the benefit of HCQ as a treatment of COVID-19^14^.

Nanoparticles encapsulating drugs or attaching to therapeutics can be utilized as drug delivery systems to change drug biodistribution, decrease toxicity, modify drug release rate, and target affected tissues or cells^15–18^. However, most nanoparticles are still in the clinical trial stage, with a few having been accepted for clinical use^16^. In this regard, noble metal nanoparticles are well-known as promising materials that can transport drugs to specific targets in the body and be engineered to develop new delivery systems^19^. Notably, silver, gold, and platinum nanoparticles reveal stability in the biological environment and survive in an intracellular environment^20–22^. The stable nanoparticles with small size possess the advantage of easily interacting with biomolecules both on the surface and inside cells, thereby playing a significant role in biomedical applications such as drug vehicles in diagnosing and treating diseases.

Noble metals, especially silver, has a long history of usage as antibacterial materials^23–26^, and current studies have utilized the antiviral and immunomodulatory properties of silver nanoparticles (AgNPs)^27,28^. Recently, Garofalo et al.^29^ demonstrated the in vivo antiviral activity of AgNPs during respiratory syncytial virus (RSV) infection. On the other hand, the biocompatibility and easy synthesis process of gold nanoparticles (AuNPs) as another noble metal, makes them appropriate candidates in the drug delivery system^30,31^, especially in cancer therapies^32,33^. Although Ag, Au, and Pt compounds are well-known for being safe for humans, the toxicity of nanoparticles should be carefully considered in nanomedicine. In this regard, previous studies have indicated that PtNP is less toxic compared to AgNPs^34^. Furthermore, different green synthesized processes have been proposed to potentially decrease the toxicity issues of nanoparticles and their side-effects in medications^35–38^. However, it is necessary to investigate their toxicity in more detail for their particular usage.

Current work is done under extended lockdown across the world, with no possibility of accessing experimental laboratories. In this situation, the work is just beginning for computational chemists and biophysicists to model the different approaches and propose efficient therapies to the experimentalists. Computational studies of molecular interactions of drugs can be used to develop the next-generation of drug inventions such as target-based drug discovery and delivery.

This paper has carried out the first principle density functional calculations to determine the affinity of HCQ/CQ molecules towards noble nanoparticles and confirm their weak interaction by theoretical UV–Vis absorption spectra. The slab model using plane wave DFT was used to demonstrate the trend in the affinity of the drug molecules towards the noble metal nanoparticles. The complementary calculations were done based on molecular dynamics simulations by changing the size and composition of metal nanoparticles and the number of coated HCQ molecules. Our computational findings on the interaction of noble nanoparticles with drugs suggest these materials as potential vehicles for efficient HCQ/CQ usage to decrease their side effects.

## Computational details

**Figure 1:**
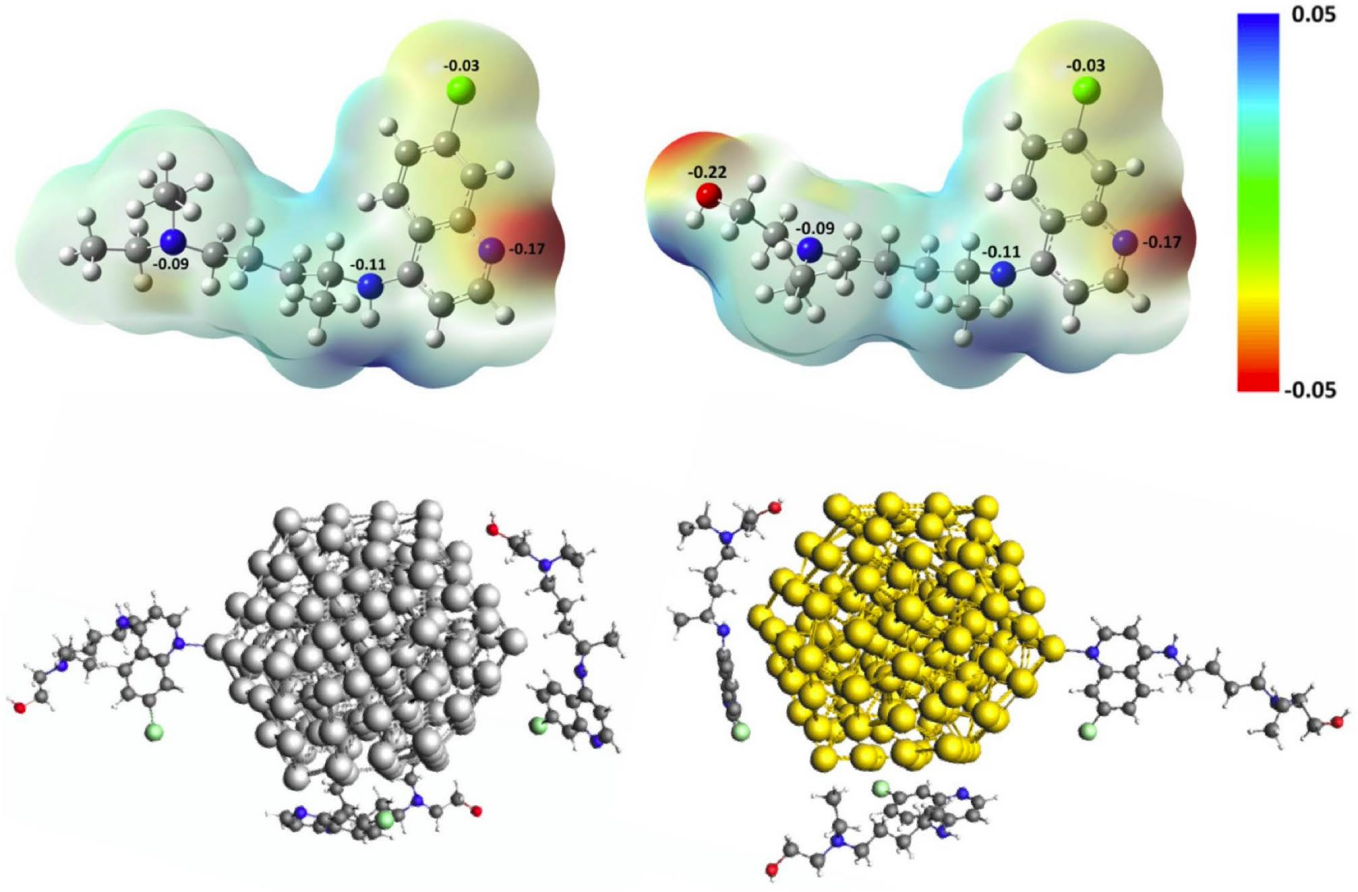
Charge distribution of HCQ and CQ molecules and their electrostatic potential map. Stable geometry of Ag_147_ and Au_147_ complexed with HCQ molecules (at BPE-D3/TZP level of theory).

For HCQ and CQ molecules, the geometry optimization and frequency calculation were performed with PBE generalized gradient (GGA) exchange–correlation (xc-) density functional^39^ with the inclusion of the Grimme dispersion correction scheme (D3)^40–42^ applying Becke-Johnson damping and a triple-$\zeta$ polarized (TZP) Slater type basis set (PBE-D3/TZP). The Conductor like Screening Model (COSMO)^43^ was considered to model the effect of water solvent. For the optimized structure, the Hirshfeld point charges^44^ and electrostatic potential map were obtained both with and without the solvent (in Fig. 1 and Supplementary Fig. S1). The experimental interatomic metal–metal distance was employed to create starting structures for further optimization with the LDA (local density approximation) xc-functional^45^ and the scalar relativistic ZORA formalism^46,47^. The interactions of HCQ with icosahedral silver and gold clusters with 147 atoms were investigated at the PBE-D3/TZP level of the theory under the influence of the relativistic effect (ZORA). To determine the effect of HCQ drug on the electronic structures and the plasmonic absorption spectra of noble metal particles, the recently developed time-dependent density functional approach, TD-DFT + TB method^48,49^ which combines a full DFT ground state with tight-binding approximations, was applied. The excited states calculations were performed at optimized geometries using the asymptotically corrected LB94 xc-functional^50^, and the absorption spectra were obtained in the range of 0.0–6.0 eV. All these calculations were performed with the Amsterdam Density Functional (ADF2019.1) program^51^.

A periodic slab model has been considered to study the adsorption of HCQ molecules on the Ag(111), Au(111), and Pt(111) surfaces using the Quantum ESPRESSO package^52^ with PBE-D3^39,40^ functional. The cut-off energy of 80 Rydberg ($\sim$ 1088 eV) was employed for the plane-wave basis set, and the electron–ion interactions were represented through the ultrasoft pseudopotential, including the scalar relativistic effects. The slab is made of four layers of Ag/Au/Pt with 36 atoms per layer. A large vacuum in the z-direction (perpendicular to the slab) was applied, and the large size of the box made it possible that just the $\Gamma$-point of the reciprocal lattice was considered. During the calculations, the first two layers were kept fixed at the bulk positions while the atoms in the top layers and molecule were allowed to relax.

The molecular dynamics (MD) simulations for interactions of HCQ and CQ with noble metal nanoparticles (NPs), Ag_147_, Au_147_, Au_92_Ag_55_, and Pt_147_ were performed in a cubic box with sides of 60 × 60 × 60 Å (structures are available in Supplementary Table S1–S6). The nanoparticles were fixed at the center of the box and were surrounded by water molecules in a random arrangement and either HCQ or CQ drugs. The time step in the simulation was 2.0 fs, and the length of time is 20 ns, under the isothermal-isobaric NPT (constant particle number, pressure, and temperature) condition at 300 K and 1 atm (controlled with Nose–Hoover algorithm). The OPLS-AA^53^ and TIP3P^54^ force fields were used to describe the interactions of drugs and water molecules, respectively. Moreover, the Lennard–Jones parameters for nanoparticles are listed in Supplementary Table S7. The electrostatic interactions were simulated with the Particle–Particle Particle-Mesh (PPPM)^55^ solver (with accuracy 1 × 10^–5^). The non-bonded dispersion interactions were computed with Lennard–Jones (LJ) 12–6 potential with the cut-off distance of 12 Å.

To study the effects of nanoparticle size, four different sizes of AgNPs with 147, 561, 1415, 2869 atoms (with diameter 1.6, 2.6, 3.6, and 4.6 nm, respectively) were placed at the simulation box with fixed (i.e., 12) and a varying number of HCQ molecules (i.e., 12, 32, 64, 105) that increased proportionally with the number of silver atoms on the surface. The simulation box was filled with 6000 water molecules. All simulations were performed using the Gromos53a6^56^ and SPC^54^ force fields. The energy was minimized by using the steepest descent minimization algorithm^57^. Equilibration of each system was done in three steps. First, an NVT ensemble coupled to the V-rescale thermal bath at 300 K was applied to the system over 100 ps. Then, an NPT ensemble coupled to the Berendsen pressure bath at 1 atm over 200 ps was applied to the system. Finally, the system was subjected to a 50 ns molecular dynamics (MD) simulation under constant conditions of 1 atm and 300 K with a time step of 1 fs. The LINCS algorithm^58^ was used to constraint the bond lengths, and the long-range electrostatics were applied using the particle mesh Ewald (PME)^59^. The LAMMPS^60^ and GROMACS packages^61,62^ were used for molecular dynamics simulations, and the VMD package^63^ was used for the visualization.

## Results and discussion

### Interaction of HCQ with AgNP and AuNP and its influence on absorption spectra

**Figure 2:**
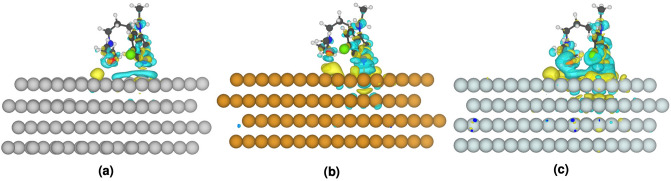
The charge density difference of adsorbed of HCQ molecules on a periodic slab model of Ag(111), Au(111), and Pt(111). The isovalue for the charge transfer plot, is fixed at 0.001e/a.u^3^ . Yellow and blue colors indicate positive and negative level corresponds to gain and loss of electron density.

The charge distribution of HCQ and CQ molecules and their electrostatic potential map in Fig. 1 displays the active sites of these molecules for interaction with noble metal NPs. The initial structure of complexes was generated by placing the small silver cluster near the electron-rich sites (e.g., N-, O- and Cl- groups). These sites can donate the electron density via their lone pairs to 4d and 5s orbitals of the silver atom^64,65^. The nitrogen of the pyridine ring in CQ and HCQ and the oxygen of the hydroxyl group in HCQ have the highest affinity for interaction with noble metal clusters. Moreover, the optimized structure of HCQ/CQ on Ag(111), Au(111), and Pt(111) layers exhibited the highest affinity of drug molecules toward the platinum surface, and their charge density difference confirmed the transfer of charge and accumulation on the metal surface (see Fig. 2, Supplementary Fig. S2, and Supplementary Table S8).

In addition, Fig. 1 shows the stable geometry of icosahedral Ag_147_ and Au_147_ nanoparticles which are complexed with HCQ molecules (at PBE-D3/TZP level of theory). Here, the non-covalent charge-transfer interactions with partially negative charge groups of the molecule play an essential role in determining the ability of nanoparticles to bond with HCQ or CQ. The binding energy of HCQ with AgNP (at PBE-D3/TZP level of theory) is about ∆E_b_ =  − 21.06 kcal mol^−1^ (per HCQ molecule), while the interaction energy with AuNP is about ∆E_b_ =  − 29.39 kcal mol^−1^ more favorable than silver. The higher electron affinity of gold (EA_Au_ = 2.31 eV) compared to silver (EA_Ag_ = 1.30 eV)^66^ gives rise to increasing the interaction energy of gold atoms towards the lone-pair of HCQ. This is also confirmed by the density difference map and accumulation of negative charge on the Au(111) surface. In this regard, the adsorption energy of HCQ towards Pt(111) is about 60% more than the Au(111) surface (see Fig. 2, Supplementary Fig. S2, and Supplementary Table S8).

**Figure 3:**
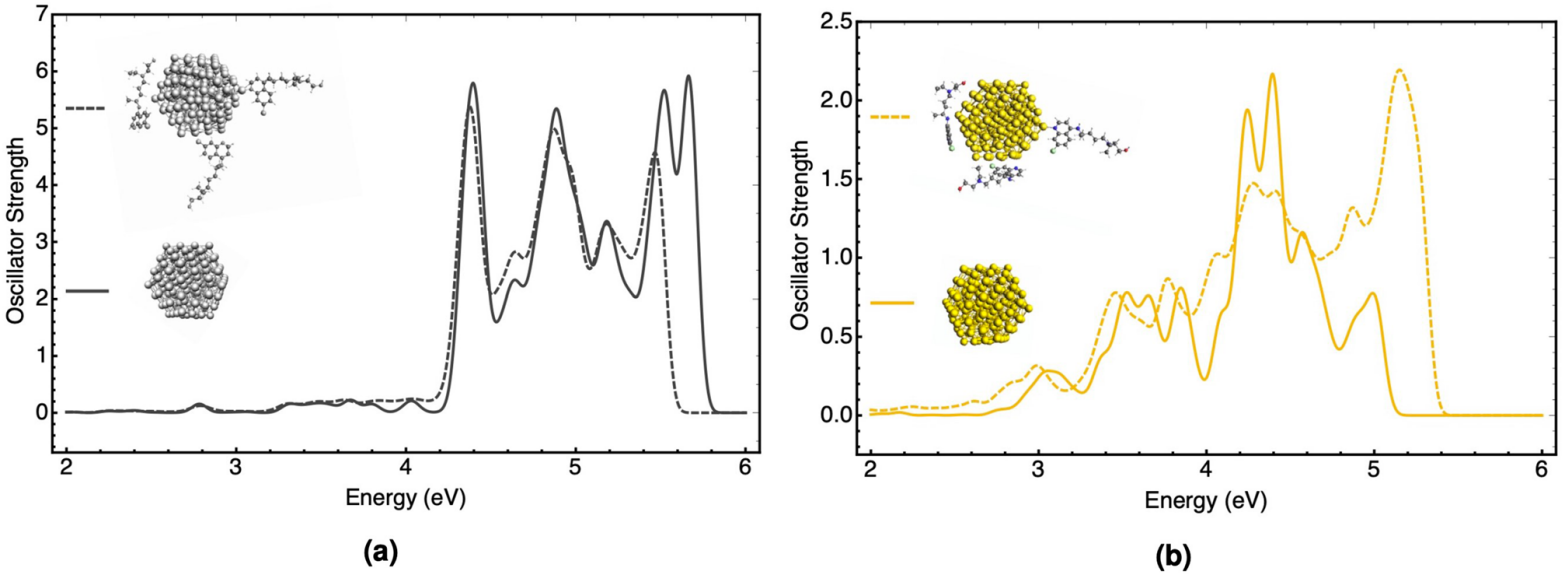
Comparison of the plasmonic absorption spectra of bare (solid line) and complexed (dash line) for **(a)** AgNP-HCQ and **(b)** AuNP-HCQ at the TD-DFT + TB level of theory. Spectra have been broadened with a σ = 0.1 eV Gaussian.

The absorption UV–Vis spectrum of bare nanoparticles and its variation under the effect of coated compounds provides further crucial evidence that can be compared with the experimental results. The spectrum can accurately estimate the effect of adsorbent molecules on the variation of electronic structures of metal NPs. Silver and gold nanoparticles are well-known for their high-intensity plasmonic absorption spectra in the UV–Vis range that can be varied by coating with the drugs. Here, the TD-DFT + TB calculations for the optimized AgNP-HCQ and AuNP-HCQ complexes compared to bare nanoparticles were obtained. TD-DFT + TB, as an accurate and efficient approach, obtained the ground-state orbitals with DFT and the excited-state properties with the tight-binding method. As shown in Fig. 3, for the AgNP-HCQ complex, the plasmonic spectrum compared to bare AgNP did not change clearly, and just the intensity of peaks around 5.5 eV damped slightly. On the other hand, for the AuNP-HCQ complex after the adsorption of HCQ, the plasmonic peak of gold in the range of 4.0–4.5 eV exhibited a distinct variation in energy and intensity. The shift of the high-intensity peak to blue established the more perturbative effect of HCQ adsorption on the electronic structure and plasmonic spectrum of AuNP in comparison to AgNP.

### AgNP, AuNP, AgAuNP, and PtNP coated with HCQ(CQ)

In this part, the effect of changing the type of nanoparticles and increasing the number of HCQ molecules on the coating properties of nanoparticles are discussed based on molecular dynamics calculations and the trend of the radial distribution function (RDF). RDF depicts how the density of one molecule changes as a function of the distance from another reference molecule. Besides, RDF can be used to represent distance-dependent relative probability for observing a given site (or atom) relative to some central site (or atom). This analysis provides the microstructure information about the arrangement of HCQ/CQ molecules and their affinity for interactions with nanoparticles^67–69^.

**Figure 4:**
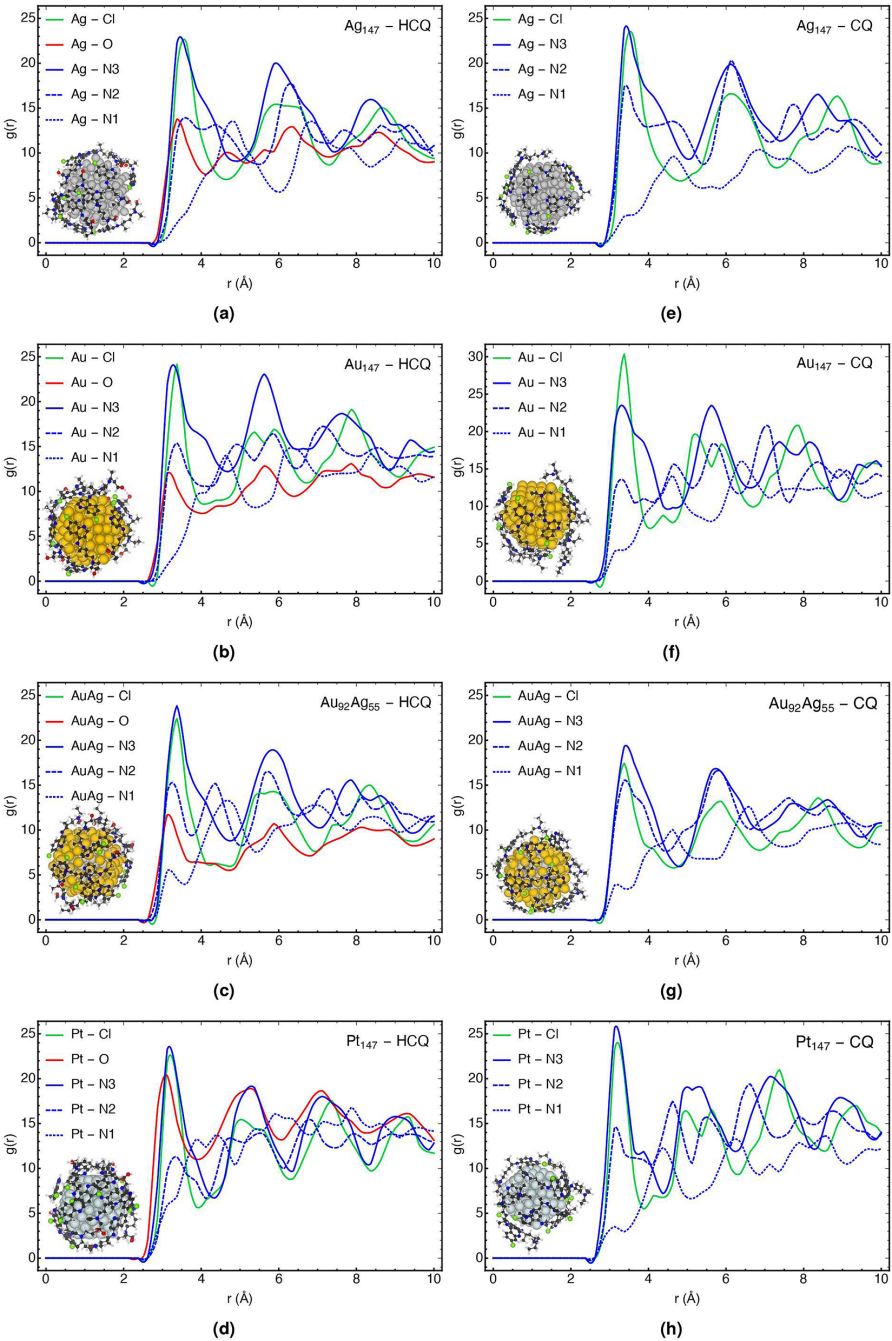
RDF plots for the active sites of HCQ/CQ with different types of (147-atomic icosahedral) nanoparticles such as AgNP, AuNP, AgAuNP, and PtNP.

Figure 4 displays the RDF, g(r), for the active sites of HCQ/CQ molecules with different types of (147-atomic icosahedral) nanoparticles such as AgNP, AuNP, AgAuNP, and PtNP. As can be found in Fig. 4, the nitrogen of the pyridine ring has the highest affinity in comparison to other types of nitrogen, which is in agreement with the DFT calculation in the previous section. However, it can be seen in Fig. 4 that the peak of chlorine increases in the same way as nitrogen, which can be related to its vicinity to the nitrogen atom of the pyridine ring and not the affinity of the Cl-group. The minimum DFT structure (in the previous section) and the lower negative charge of Cl (− 0.03 $|e|$) in comparison to N (− 0.17 $|e|$) further strengthen this claim. In addition, the coating of Ag_147_ with more HCQ molecules was simulated (in Supplementary Fig. S6a), which confirmed higher g(r) for N-group with respect to Cl-group. The probability distribution map of atoms near the nanoparticles (see Supplementary Fig. S3-S4 in supporting information) confirms the RDF results.

Furthermore, the O-atom of the hydroxyl group is another active site of HCQ for interaction. However, for gold, silver, and alloy nanoparticles, the g(r) values for O-group is lower than N-group, and for PtNP, it is slightly more than N-group. For PtNP–HCQ in Fig. 4d, the approximately similar RDF peaks around 3.0 Å for both O-group and N-group can be related to the high affinity of the PtNP to interact with both sides of HCQ. For gold and silver noble metals, the higher attraction of N-group (verse O-group) was established by Antusek et al. based on ab-initio calculation^70^.

**Figure 5:**
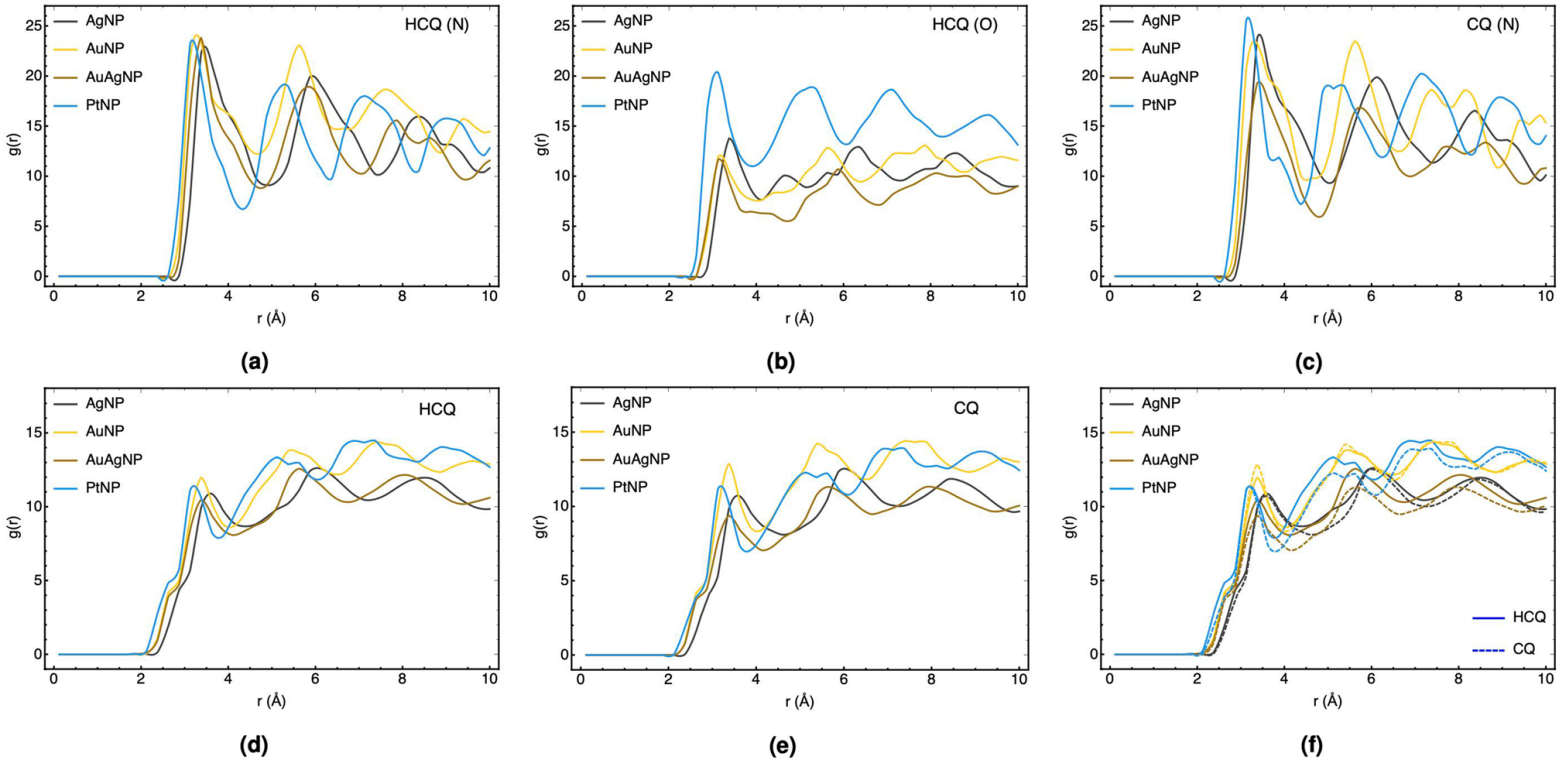
Comparison of the total and atom type RDF plots for HCQ/CQ molecules with AgNP, AuNP, AgAuNP, and PtNP.

Figure 5 compares the total and atom type RDFs of HCQ/CQ molecules with respect to the variation of nanoparticles to propose the possible candidates for adsorption. Figure 5a–c compares the RDF of N- and O-atom of HCQ and N-atom of CQ with respect to the type of nanoparticles. For N-group, the affinity of different types of nanoparticles is nearly similar to each other, although PtNP has numerous robust peaks in the range of $<$ 10 Å. On the other hand, the sharp and intense g(r) peak for O-group with PtNP, which appears at a shorter distance, demonstrates the best adsorption properties on PtNP and explains the stronger affinity of PtNP to HCQ compared to CQ. Accordingly, we can conclude that HCQ preferred to adsorb on AuNP, AgNP, and alloy from one side and coated on PtNP by N- and O-group.

Finally, Fig. 5d,e show the total RDF plots of HCQ and CQ molecules with respect to the type of nanoparticles and demonstrated that the overall coating trend is as follows: PtNP $>$ AuNP $>$ AuAgNP $>$ AgNP. Additionally, comparing the total RDF of NPs with HCQ and CQ (Fig. 5f) indicates that PtNP has a higher g(r) and more affinity towards HCQ than CQ, which is in agreement with our DFT results presented in Supplementary Table S8.

### Coated HCQ on the different size of Ag_n_ (n = 147, 561, 1415, and 2869)

In this part, the effect of the size on the adsorption properties of AgNPs is investigated. The calculated diameters of Ag_147_, Ag_561_, Ag_1415,_ and Ag_2869_ nanoparticles are 1.6, 2.6, 3.6, and 4.6 nm, respectively, while their thickness after coating with HCQ increases about 1 nm. The AgNPs are coated with the fixed number of HCQ (12 molecules) interacting with the twelve active sites in their corners. In another attempt, the AgNPs with different sizes are coated with 12, 32, 64, and 105 molecules, proportional to the numbers of their surface atoms.

As can be found in Supplementary Fig. S5, coating nanoparticles with HCQ molecules exhibit significant fluctuations at the beginning of the simulations, indicating the free movement of HCQ molecules near the nanoparticles due to the spatial setting of the medicines in the active site. However, after ~ 5 ns, the fluctuations disappear, maintaining a continuous equilibrium to the end of the simulation time. These results suggest the reliable stabilities of the dynamic equilibriums for the complexes and their trajectories could be useful in collecting snapshots for further analyses (see Supplementary Fig. S5).

**Figure 6:**
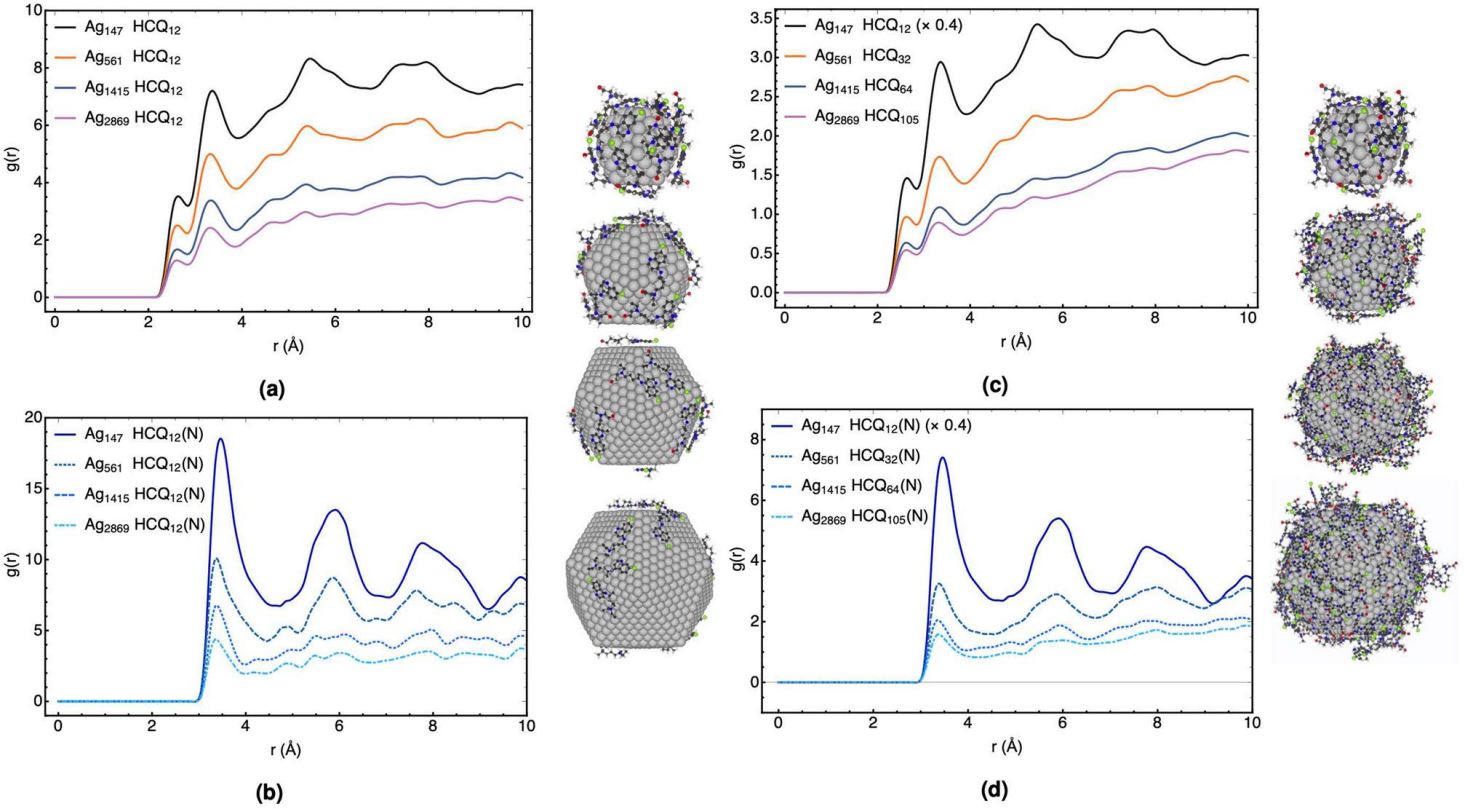
RDF of HCQ with different size of AgNPs. **(a,b)** AgNPs are coated with the same number of HCQ molecules. **(c,d)** AgNPs are coated with the different number of HCQ molecules.

In Fig. 6 (and Supplementary Fig. S6 and S7), the RDF plots for the fixed and varying numbers of HCQ with respect to the size of AgNPs and the type of anchoring atoms are displayed. The comparison of the RDF plots for the N-group as an active-site (Fig. 6b), in addition to all atoms (Fig. 6a), exhibits a decreasing trend in the adsorption properties by going from small to larger AgNPs. The same trend was reproduced in Fig. 6c,d, in which the portion of HCQs increases with respect to the number of atoms on the surface. Generally, the overall coating properties decrease as the size of nanoparticles increases from 1.6 to 4.6 nm (see Supplementary Fig. S8). It has been established that the affinity of noble metal clusters is lowered by increasing their size. Moreover, in agreement with the previous section, the N-group has a higher affinity for interaction with different size of AgNPs with respect to the O-group (see Supplementary Fig. S6 and S7). Finally, Supplementary Fig. S8 shows the comparison of RDF for HCQ and CQ coated on Ag_2869_ and reveals the appropriate adsorption affinity of HCQ versus CQ.

## Conclusion

In summary, the adsorption and the coating properties of noble metal nanoparticles with HCQ/CQ molecules have been studied as a potentially efficient strategy for in vivo usage of these drugs. The weak charge-transfer interaction with partially negative charge groups of drugs was investigated, and it was established by changing the type of nanoparticle elements that the affinity towards N and O groups increases as follows: AgNP < AuAgNP < AuNP < PtNP. Following the investigation of the effect of size on the coating properties, it was found that the overall affinity decreases by increasing the size from 1.6 nm (for Ag_147_) to 2.6 nm (for Ag_561_). For Ag_561_, Ag_1415,_ and Ag_2869_ with diameters 2.6 to 4.6 nm, a nearly similar decrease in affinity was obtained. Finally, based on the quantum mechanics and molecular dynamics simulation, we can suggest these noble nanoparticles (with low toxicity and antiviral activity) as appropriate vehicles for efficient HCQ/CQ usage with decreased side effects of the drugs.

## Supplementary Information

Supplementary Information.

## Data Availability

No datasets were generated or analyzed during the current study.
