# Supplementary material for: First principle simulation of coated hydroxychloroquine on Ag, Au and Pt nanoparticles
Source: Sci Rep. 2021 Jan 22;11:2131. doi: 10.1038/s41598-021-81617-6 (PMC7822900; doi:10.1038/s41598-021-81617-6)
Supplement: Supplementary file 1 — Supplementary Information. [file 41598_2021_81617_MOESM1_ESM.pdf]

Supplementary information: First Principle Simulation of Coated Hydroxychloroquine on Ag, Au and Pt Nanoparticles

Razieh Morad[a,b,1], Mahmood Akbari[a,b,1], Parham Rezaee[c,1], Amin Koochaki[c,1], Malik Maaza[*a,b], and Zahra Jamshidi[*c]

[a]UNESCO-UNISA Africa Chair in Nanoscience & Nanotechnology (U2ACN2), College of Graduate Studies, University of South Africa (UNISA), Pretoria, South Africa
[b]Nanoscience African Network (NANOAFNET), Material Research Division, iThemba LABS-National Research Foundation, Somerset West 7129, South Africa
[c]Chemistry Department, Sharif University of Technology, Tehran 11155-9516, Iran
[*]maaza@tlabs.ac.za
[*]njamshidi@sharif.edu
[1]these authors contributed equally to this work
The authors dedicate this study to healthcare workers for their sacrifices in the pandemic.

ABSTRACT

This provide Supplementary Information on the First Principle Simulation of Coated Hydroxychloroquine on Ag, Au and Pt Nanoparticles article.

List of Tables

S1 Optimized geometry of CQ structure with density functional theory at PBE-D3/TZP level of theory. 2
S2 Optimized geometry of HCQ structure with density functional theory at PBE-D3/TZP level of theory. 3
S3 Optimized geometry of Ag_{147} structure with density functional theory at LDA/DZ level of theory. 4
S4 Optimized geometry of Au_{147} structure with density functional theory at LDA/DZ level of theory. 7
S5 Optimized geometry of $Au_{92}Ag_{55}$ structure with density functional theory at LDA/DZ level of theory. 10
S6 Optimized geometry of Pt_{147} structure with density functional theory at LDA/DZ level of theory. 13
S7 The Lennard-Jones parameters for nanoparticles simulation. 16
S8 The interaction energy of HCQ and CQ molecules with 2-layer slab model of Ag (111), Au (111), and Pt (111). . 16

List of Figures

S1 Charge distribution of HCQ and CQ molecules and their electrostatic potential map (Iso–value = 0.0004 $e\text{Å}^{-3}$). . 17
S2 The charge density difference of Adsorbed of HCQ /CQ molecules on a periodic slab model of the Ag(111), Au(111), and Pt(111). The isovalue for the charge transfer plot, is fixed at $0.001 e/a.u^3$. Yellow and blue color indicate positive and negative level corresponds to gain and loss of electron density. 18
S3 Probability distribution of Cl, N (in pyridine ring) and O of HCQ near the (a–c) AgNP, (d–f) AuNP, (g–i) AuAgNP, and (j–l) PtNP. 19
S4 Probability distribution of Cl and N (in pyridine ring) of CQ near the (a,b) AgNP, (c,d) AuNP, (e,f) AuAgNP, and (g,h) PtNP. 20
S5 RMSD of different size of AgNPs coated with HCQs. (a) fixed number of HCQs (12) (b) increasing number of HCQ proportional to the Ag atom on the surface of NPs. 21
S6 The comparison of RDFs for (a) Ag_{147}, (b) Ag_{561}, (c) Ag_{1415} and (d) Ag_{2869} with 12 molecules of HCQ. 22
S7 The comparison of RDFs for (a) Ag_{147}, (b) Ag_{561}, (c) Ag_{1415} and (d) Ag_{2869} with 12, 32, 64 and 105 molecules of HCQ. 23
S8 Decreasing the overall coating properties by increasing the size of AgNP from 1.6 (Ag_{147}) to 4.6 (Ag_{2869}) nm. The mean values of RDF are calculated using Eq. S1 and S2. 24
S9 The comparison of RDFs for HCQ and CQ with Ag_{2869}. 25

Table S1. Optimized geometry of CQ structure with density functional theory at PBE-D3/TZP level of theory.

	atom	x	y	z	q
1	N	3.5332	2.2412	-1.7211	-0.1145
2	C	4.6379	2.9236	-1.0203	0.0227
3	C	2.2618	2.4899	-1.0838	0.0549
4	C	5.9551	2.2034	-1.3744	-0.0694
5	C	4.6948	4.4185	-1.3896	-0.1147
6	C	1.8341	1.7165	-0.0580	-0.0163
7	C	1.3666	3.5667	-1.5925	-0.0745
8	C	7.1267	2.6107	-0.4660	-0.0677
9	C	0.5095	1.9974	0.5588	0.0438
10	C	2.6265	0.5859	0.4915	-0.0415
11	C	0.1769	3.7785	-1.0066	0.0111
12	C	8.3683	1.7507	-0.7476	-0.0268
13	N	-0.2405	2.9619	0.1031	-0.1710
14	C	0.0586	1.1638	1.6912	-0.0573
15	C	2.1532	-0.1353	1.5226	-0.0582
16	N	9.5291	2.2234	0.0415	-0.0891
17	C	0.8339	0.1621	2.1439	0.0151
18	C	9.4037	1.8625	1.4783	-0.0243
19	C	10.7940	1.7213	-0.5423	-0.0264
20	Cl	0.2830	-0.8216	3.4917	-0.0322
21	C	9.3535	3.1314	2.3205	-0.1169
22	C	11.9893	2.5358	-0.0482	-0.1141
23	H	3.4952	2.5430	-2.7232	0.1127
24	H	4.4912	2.8420	0.0816	0.0385
25	H	6.2171	2.3985	-2.4378	0.0350
26	H	5.7937	1.1078	-1.2645	0.0346
27	H	5.4907	4.9357	-0.8164	0.0446
28	H	3.7385	4.9171	-1.1326	0.0409
29	H	4.8879	4.5489	-2.4757	0.0374
30	H	1.6720	4.1824	-2.4329	0.0458
31	H	6.8199	2.4941	0.5947	0.0354
32	H	7.3903	3.6747	-0.6428	0.0361
33	H	3.5925	0.3282	0.0685	0.0476
34	H	-0.4768	4.5641	-1.3709	0.0480
35	H	8.5802	1.8377	-1.8370	0.0377
36	H	8.1571	0.6776	-0.5363	0.0211
37	H	-0.9027	1.3628	2.1561	0.0530
38	H	2.7470	-0.9568	1.9115	0.0492
39	H	10.2523	1.2383	1.8373	0.0335
40	H	8.4883	1.2660	1.6906	0.0312
41	H	10.7828	1.8255	-1.6500	0.0383
42	H	10.9414	0.6421	-0.3115	0.0217
43	H	10.2834	3.7217	2.1812	0.0372
44	H	9.2562	2.8654	3.3942	0.0375
45	H	8.4828	3.7541	2.0261	0.0353
46	H	12.9045	2.2203	-0.5922	0.0409
47	H	12.1595	2.3762	1.0364	0.0365
48	H	11.8172	3.6168	-0.2366	0.0384

Table S2. Optimized geometry of HCQ structure with density functional theory at PBE-D3/TZP level of theory.

	atom	x	y	z	q
1	N	0.0000	0.0000	0.0000	-0.1153
2	C	1.4710	0.0000	0.0000	0.0227
3	C	-0.4900	0.0000	1.3870	0.0548
4	C	1.9810	0.0000	-1.4430	-0.0687
5	C	1.9610	1.2500	0.7360	-0.1151
6	C	-0.7210	-1.2000	2.0400	-0.0162
7	C	-0.7210	1.2000	2.0400	-0.0741
8	C	3.5120	0.0000	-1.4430	-0.0674
9	C	-1.1840	-1.2000	3.3470	0.0439
10	C	-0.4900	-2.4010	1.3870	-0.0413
11	C	-1.1840	1.2000	3.3470	0.0113
12	C	4.0230	0.0000	-2.8870	-0.0256
13	N	-1.4150	0.0000	4.0010	-0.1713
14	C	-1.4150	-2.4010	4.0010	-0.0571
15	C	-0.7210	-3.6010	2.0400	-0.0574
16	N	5.4940	0.0000	-2.8870	-0.0867
17	C	-1.1840	-3.6010	3.3470	0.0155
18	C	5.9840	0.0000	-4.2740	-0.0372
19	C	5.9840	1.2010	-2.1930	-0.0241
20	Cl	-1.4790	-5.1360	4.1820	-0.0312
21	C	7.5150	0.0000	-4.2740	-0.0056
22	C	6.1540	0.8960	-0.7030	-0.1164
23	O	7.9850	0.0000	-5.6030	-0.2169
24	H	-0.3370	0.8260	-0.4770	0.1123
25	H	1.8120	-0.9060	0.5130	0.0390
26	H	1.6160	-0.8950	-1.9600	0.0364
27	H	1.4850	0.6790	-2.1460	0.0340
28	H	2.0780	1.0240	1.8020	0.0412
29	H	2.9600	1.6030	0.4560	0.0437
30	H	1.4270	2.2070	0.6920	0.0370
31	H	-0.5390	2.1500	1.5240	0.0458
32	H	3.8780	0.8950	-0.9270	0.0336
33	H	4.0090	-0.6790	-0.7410	0.0385
34	H	-0.1250	-2.4010	0.3540	0.0480
35	H	-1.3660	2.1500	3.8640	0.0481
36	H	3.6720	-0.8950	-3.4140	0.0396
37	H	3.5520	0.6910	-3.5950	0.0220
38	H	-1.7800	-2.4010	5.0340	0.0532
39	H	-0.5390	-4.5510	1.5240	0.0498
40	H	5.6040	-0.8950	-4.7800	0.0408
41	H	5.4740	0.6910	-4.9540	0.0188
42	H	6.9450	1.4920	-2.6340	0.0329
43	H	5.4890	2.1390	-2.4690	0.0315
44	H	7.9100	0.8950	-3.7790	0.0306
45	H	8.0390	-0.7040	-3.6170	0.0439
46	H	6.5190	1.7910	-0.1860	0.0385
47	H	5.2310	0.6670	-0.1580	0.0363
48	H	6.7740	0.0700	-0.3370	0.0378
49	H	7.3630	0.0000	-6.3310	0.1461

Table S3. Optimized geometry of Ag_{147} structure with density functional theory at LDA/DZ level of theory.

	atom	x	y	z
1	Ag	0.000	0.000	0.000
2	Ag	1.442	1.985	1.227
3	Ag	0.000	0.000	2.744
4	Ag	2.334	-0.758	1.227
5	Ag	1.442	-1.985	-1.227
6	Ag	2.334	0.758	-1.227
7	Ag	0.000	2.454	-1.227
8	Ag	-1.442	1.985	1.227
9	Ag	0.000	-2.454	1.227
10	Ag	0.000	0.000	-2.744
11	Ag	-2.334	-0.758	1.227
12	Ag	-1.442	-1.985	-1.227
13	Ag	-2.334	0.758	-1.227
14	Ag	2.885	3.971	2.454
15	Ag	0.000	0.000	5.488
16	Ag	4.668	-1.517	2.454
17	Ag	2.885	-3.971	-2.454
18	Ag	4.668	1.517	-2.454
19	Ag	0.000	4.908	-2.454
20	Ag	-2.885	3.971	2.454
21	Ag	0.000	-4.908	2.454
22	Ag	0.000	0.000	-5.488
23	Ag	-4.668	-1.517	2.454
24	Ag	-2.885	-3.971	-2.454
25	Ag	-4.668	1.517	-2.454
26	Ag	1.442	1.985	3.971
27	Ag	3.776	1.227	2.454
28	Ag	3.776	2.744	0.000
29	Ag	1.442	4.440	0.000
30	Ag	0.000	3.971	2.454
31	Ag	0.000	-3.971	-2.454
32	Ag	-1.442	-4.440	0.000
33	Ag	-1.442	-1.985	-3.971
34	Ag	-3.776	-2.744	0.000
35	Ag	-3.776	-1.227	-2.454
36	Ag	2.334	-0.758	3.971
37	Ag	-1.442	1.985	3.971
38	Ag	0.000	-2.454	3.971
39	Ag	-2.334	-0.758	3.971
40	Ag	1.442	-1.985	-3.971
41	Ag	2.334	0.758	-3.971
42	Ag	0.000	2.454	-3.971
43	Ag	-2.334	0.758	-3.971
44	Ag	3.776	-2.744	0.000
45	Ag	4.668	0.000	0.000
46	Ag	2.334	-3.213	2.454
47	Ag	-2.334	3.213	-2.454
48	Ag	-3.776	2.744	0.000
49	Ag	-4.668	0.000	0.000
50	Ag	-1.442	4.440	0.000
51	Ag	-3.776	1.227	2.454
52	Ag	3.776	-1.227	-2.454

53	Ag	1.442	-4.440	0.000
54	Ag	-2.334	-3.213	2.454
55	Ag	2.334	3.213	-2.454
56	Ag	4.327	5.956	3.681
57	Ag	0.000	0.000	8.231
58	Ag	7.002	-2.275	3.681
59	Ag	4.327	-5.956	-3.681
60	Ag	7.002	2.275	-3.681
61	Ag	0.000	7.362	-3.681
62	Ag	-4.327	5.956	3.681
63	Ag	0.000	-7.362	3.681
64	Ag	0.000	0.000	-8.231
65	Ag	-7.002	-2.275	3.681
66	Ag	-4.327	-5.956	-3.681
67	Ag	-7.002	2.275	-3.681
68	Ag	2.885	3.971	5.198
69	Ag	1.442	1.985	6.715
70	Ag	5.219	3.213	3.681
71	Ag	6.110	0.469	3.681
72	Ag	5.219	4.729	1.227
73	Ag	6.110	3.502	-1.227
74	Ag	2.885	6.425	1.227
75	Ag	1.442	6.894	-1.227
76	Ag	1.442	5.956	3.681
77	Ag	-1.442	5.956	3.681
78	Ag	-1.442	-5.956	-3.681
79	Ag	1.442	-5.956	-3.681
80	Ag	-2.885	-6.425	-1.227
81	Ag	-1.442	-6.894	1.227
82	Ag	-2.885	-3.971	-5.198
83	Ag	-1.442	-1.985	-6.715
84	Ag	-5.219	-4.729	-1.227
85	Ag	-6.110	-3.502	1.227
86	Ag	-5.219	-3.213	-3.681
87	Ag	-6.110	-0.469	-3.681
88	Ag	2.334	-0.758	6.715
89	Ag	4.668	-1.517	5.198
90	Ag	-1.442	1.985	6.715
91	Ag	-2.885	3.971	5.198
92	Ag	0.000	-2.454	6.715
93	Ag	0.000	-4.908	5.198
94	Ag	-2.334	-0.758	6.715
95	Ag	-4.668	-1.517	5.198
96	Ag	1.442	-1.985	-6.715
97	Ag	2.885	-3.971	-5.198
98	Ag	2.334	0.758	-6.715
99	Ag	4.668	1.517	-5.198
100	Ag	0.000	2.454	-6.715
101	Ag	0.000	4.908	-5.198
102	Ag	-2.334	0.758	-6.715
103	Ag	-4.668	1.517	-5.198
104	Ag	6.110	-3.502	1.227
105	Ag	5.219	-4.729	-1.227
106	Ag	7.002	-0.758	1.227
107	Ag	7.002	0.758	-1.227

108	Ag	4.668	-3.971	3.681
109	Ag	2.334	-5.667	3.681
110	Ag	-4.668	3.971	-3.681
111	Ag	-2.334	5.667	-3.681
112	Ag	-6.110	3.502	-1.227
113	Ag	-5.219	4.729	1.227
114	Ag	-7.002	0.758	-1.227
115	Ag	-7.002	-0.758	1.227
116	Ag	-2.885	6.425	1.227
117	Ag	-1.442	6.894	-1.227
118	Ag	-5.219	3.213	3.681
119	Ag	-6.110	0.469	3.681
120	Ag	5.219	-3.213	-3.681
121	Ag	6.110	-0.469	-3.681
122	Ag	2.885	-6.425	-1.227
123	Ag	1.442	-6.894	1.227
124	Ag	-2.334	-5.667	3.681
125	Ag	-4.668	-3.971	3.681
126	Ag	2.334	5.667	-3.681
127	Ag	4.668	3.971	-3.681
128	Ag	3.776	1.227	5.198
129	Ag	6.110	1.985	1.227
130	Ag	3.776	5.198	-1.227
131	Ag	0.000	6.425	1.227
132	Ag	0.000	3.971	5.198
133	Ag	0.000	-6.425	-1.227
134	Ag	0.000	-3.971	-5.198
135	Ag	-3.776	-1.227	-5.198
136	Ag	-6.110	-1.985	-1.227
137	Ag	-3.776	-5.198	1.227
138	Ag	2.334	-3.213	5.198
139	Ag	-2.334	-3.213	5.198
140	Ag	-3.776	1.227	5.198
141	Ag	-2.334	3.213	-5.198
142	Ag	2.334	3.213	-5.198
143	Ag	3.776	-1.227	-5.198
144	Ag	3.776	-5.198	1.227
145	Ag	6.110	-1.985	-1.227
146	Ag	-3.776	5.198	-1.227
147	Ag	-6.110	1.985	1.227

Table S4. Optimized geometry of Au_{147} structure with density functional theory at LDA/DZ level of theory.

	atom	x	y	z
1	Au	0.000	0.000	0.000
2	Au	1.985	1.442	1.227
3	Au	0.000	0.000	2.744
4	Au	1.985	-1.442	1.227
5	Au	0.758	-2.334	-1.227
6	Au	2.454	0.000	-1.227
7	Au	0.758	2.334	-1.227
8	Au	-0.758	2.334	1.227
9	Au	-0.758	-2.334	1.227
10	Au	0.000	0.000	-2.744
11	Au	-2.454	0.000	1.227
12	Au	-1.985	-1.442	-1.227
13	Au	-1.985	1.442	-1.227
14	Au	3.971	2.885	2.454
15	Au	0.000	0.000	5.488
16	Au	3.971	-2.885	2.454
17	Au	1.517	-4.668	-2.454
18	Au	4.908	0.000	-2.454
19	Au	1.517	4.668	-2.454
20	Au	-1.517	4.668	2.454
21	Au	-1.517	-4.668	2.454
22	Au	0.000	0.000	-5.488
23	Au	-4.908	0.000	2.454
24	Au	-3.971	-2.885	-2.454
25	Au	-3.971	2.885	-2.454
26	Au	1.985	1.442	3.971
27	Au	3.971	0.000	2.454
28	Au	4.440	1.442	0.000
29	Au	2.744	3.777	0.000
30	Au	1.227	3.777	2.454
31	Au	-1.227	-3.777	-2.454
32	Au	-2.744	-3.777	0.000
33	Au	-1.985	-1.442	-3.971
34	Au	-4.440	-1.442	0.000
35	Au	-3.971	0.000	-2.454
36	Au	1.985	-1.442	3.971
37	Au	-0.758	2.334	3.971
38	Au	-0.758	-2.334	3.971
39	Au	-2.454	0.000	3.971
40	Au	0.758	-2.334	-3.971
41	Au	2.454	0.000	-3.971
42	Au	0.758	2.334	-3.971
43	Au	-1.985	1.442	-3.971
44	Au	2.744	-3.777	0.000
45	Au	4.440	-1.442	0.000
46	Au	1.227	-3.777	2.454
47	Au	-1.227	3.777	-2.454
48	Au	-2.744	3.777	0.000
49	Au	-4.440	1.442	0.000
50	Au	0.000	4.668	0.000
51	Au	-3.212	2.334	2.454
52	Au	3.212	-2.334	-2.454

53	Au	0.000	-4.668	0.000
54	Au	-3.212	-2.334	2.454
55	Au	3.212	2.334	-2.454
56	Au	5.956	4.327	3.681
57	Au	0.000	0.000	8.231
58	Au	5.956	-4.327	3.681
59	Au	2.275	-7.002	-3.681
60	Au	7.362	0.000	-3.681
61	Au	2.275	7.002	-3.681
62	Au	-2.275	7.002	3.681
63	Au	-2.275	-7.002	3.681
64	Au	0.000	0.000	-8.231
65	Au	-7.362	0.000	3.681
66	Au	-5.956	-4.327	-3.681
67	Au	-5.956	4.327	-3.681
68	Au	3.971	2.885	5.198
69	Au	1.985	1.442	6.715
70	Au	5.956	1.442	3.681
71	Au	5.956	-1.442	3.681
72	Au	6.425	2.885	1.227
73	Au	6.894	1.442	-1.227
74	Au	4.729	5.219	1.227
75	Au	3.502	6.111	-1.227
76	Au	3.212	5.219	3.681
77	Au	0.469	6.111	3.681
78	Au	-3.212	-5.219	-3.681
79	Au	-0.469	-6.111	-3.681
80	Au	-4.729	-5.219	-1.227
81	Au	-3.502	-6.111	1.227
82	Au	-3.971	-2.885	-5.198
83	Au	-1.985	-1.442	-6.715
84	Au	-6.425	-2.885	-1.227
85	Au	-6.894	-1.442	1.227
86	Au	-5.956	-1.442	-3.681
87	Au	-5.956	1.442	-3.681
88	Au	1.985	-1.442	6.715
89	Au	3.971	-2.885	5.198
90	Au	-0.758	2.334	6.715
91	Au	-1.517	4.668	5.198
92	Au	-0.758	-2.334	6.715
93	Au	-1.517	-4.668	5.198
94	Au	-2.454	0.000	6.715
95	Au	-4.908	0.000	5.198
96	Au	0.758	-2.334	-6.715
97	Au	1.517	-4.668	-5.198
98	Au	2.454	0.000	-6.715
99	Au	4.908	0.000	-5.198
100	Au	0.758	2.334	-6.715
101	Au	1.517	4.668	-5.198
102	Au	-1.985	1.442	-6.715
103	Au	-3.971	2.885	-5.198
104	Au	4.729	-5.219	1.227
105	Au	3.502	-6.111	-1.227
106	Au	6.425	-2.885	1.227
107	Au	6.894	-1.442	-1.227

108	Au	3.212	-5.219	3.681
109	Au	0.469	-6.111	3.681
110	Au	-3.212	5.219	-3.681
111	Au	-0.469	6.111	-3.681
112	Au	-4.729	5.219	-1.227
113	Au	-3.502	6.111	1.227
114	Au	-6.425	2.885	-1.227
115	Au	-6.894	1.442	1.227
116	Au	-0.758	7.002	1.227
117	Au	0.758	7.002	-1.227
118	Au	-3.971	4.668	3.681
119	Au	-5.667	2.334	3.681
120	Au	3.971	-4.668	-3.681
121	Au	5.667	-2.334	-3.681
122	Au	0.758	-7.002	-1.227
123	Au	-0.758	-7.002	1.227
124	Au	-3.971	-4.668	3.681
125	Au	-5.667	-2.334	3.681
126	Au	3.971	4.668	-3.681
127	Au	5.667	2.334	-3.681
128	Au	3.971	0.000	5.198
129	Au	6.425	0.000	1.227
130	Au	5.198	3.777	-1.227
131	Au	1.985	6.111	1.227
132	Au	1.227	3.777	5.198
133	Au	-1.985	-6.111	-1.227
134	Au	-1.227	-3.777	-5.198
135	Au	-3.971	0.000	-5.198
136	Au	-6.425	0.000	-1.227
137	Au	-5.198	-3.777	1.227
138	Au	1.227	-3.777	5.198
139	Au	-3.212	-2.334	5.198
140	Au	-3.212	2.334	5.198
141	Au	-1.227	3.777	-5.198
142	Au	3.212	2.334	-5.198
143	Au	3.212	-2.334	-5.198
144	Au	1.985	-6.111	1.227
145	Au	5.198	-3.777	-1.227
146	Au	-1.985	6.111	-1.227
147	Au	-5.198	3.777	1.227

Table S5. Optimized geometry of $Au_{92}Ag_{55}$ structure with density functional theory at LDA/DZ level of theory.

	atom	x	y	z
1	Ag	0.000	0.000	0.000
2	Ag	-0.758	2.334	1.227
3	Ag	0.758	-2.334	-1.227
4	Ag	0.758	2.334	-1.227
5	Ag	-0.758	-2.334	1.227
6	Ag	1.985	-1.442	1.227
7	Ag	-1.985	1.442	-1.227
8	Ag	-1.985	-1.442	-1.227
9	Ag	2.454	0.000	-1.227
10	Ag	0.000	0.000	2.744
11	Ag	1.985	1.442	1.227
12	Ag	0.000	0.000	-2.744
13	Ag	-2.454	0.000	1.227
14	Ag	-4.908	0.000	2.454
15	Ag	-2.454	0.000	3.971
16	Ag	-3.213	-2.334	2.454
17	Ag	-1.517	-4.668	2.454
18	Ag	0.000	-4.668	0.000
19	Ag	1.517	-4.668	-2.454
20	Ag	3.213	-2.334	-2.454
21	Ag	0.758	-2.334	-3.971
22	Ag	0.758	2.334	-3.971
23	Ag	-1.227	3.776	-2.454
24	Ag	-4.440	1.442	0.000
25	Ag	-4.440	-1.442	0.000
26	Ag	2.744	-3.776	0.000
27	Ag	3.971	0.000	2.454
28	Ag	1.985	-1.442	3.971
29	Ag	0.000	4.668	0.000
30	Ag	-2.744	3.776	0.000
31	Ag	-0.758	-2.334	3.971
32	Ag	-0.758	2.334	3.971
33	Ag	-1.517	4.668	2.454
34	Ag	1.227	3.776	2.454
35	Ag	4.440	-1.442	0.000
36	Ag	4.440	1.442	0.000
37	Ag	1.985	1.442	3.971
38	Ag	-2.744	-3.776	0.000
39	Ag	-1.227	-3.776	-2.454
40	Ag	0.000	0.000	-5.488
41	Ag	-1.985	-1.442	-3.971
42	Ag	-3.213	2.334	2.454
43	Ag	2.744	3.776	0.000
44	Ag	0.000	0.000	5.488
45	Ag	1.227	-3.776	2.454
46	Ag	3.213	2.334	-2.454
47	Ag	4.908	0.000	-2.454
48	Ag	-3.971	0.000	-2.454
49	Ag	1.517	4.668	-2.454
50	Ag	2.454	0.000	-3.971
51	Ag	3.971	-2.885	2.454
52	Ag	3.971	2.885	2.454

53	Ag	-3.971	-2.885	-2.454
54	Ag	-1.985	1.442	-3.971
55	Ag	-3.971	2.885	-2.454
56	Au	5.956	4.327	3.681
57	Au	0.000	0.000	8.231
58	Au	5.956	-4.327	3.681
59	Au	2.275	-7.002	-3.681
60	Au	7.362	0.000	-3.681
61	Au	2.275	7.002	-3.681
62	Au	-2.275	7.002	3.681
63	Au	-2.275	-7.002	3.681
64	Au	0.000	0.000	-8.231
65	Au	-7.362	0.000	3.681
66	Au	-5.956	-4.327	-3.681
67	Au	-5.956	4.327	-3.681
68	Au	3.971	2.885	5.198
69	Au	1.985	1.442	6.715
70	Au	5.956	1.442	3.681
71	Au	5.956	-1.442	3.681
72	Au	6.425	2.885	1.227
73	Au	6.894	1.442	-1.227
74	Au	4.729	5.219	1.227
75	Au	3.502	6.110	-1.227
76	Au	3.213	5.219	3.681
77	Au	0.469	6.110	3.681
78	Au	-3.213	-5.219	-3.681
79	Au	-0.469	-6.110	-3.681
80	Au	-4.729	-5.219	-1.227
81	Au	-3.502	-6.110	1.227
82	Au	-3.971	-2.885	-5.198
83	Au	-1.985	-1.442	-6.715
84	Au	-6.425	-2.885	-1.227
85	Au	-6.894	-1.442	1.227
86	Au	-5.956	-1.442	-3.681
87	Au	-5.956	1.442	-3.681
88	Au	1.985	-1.442	6.715
89	Au	3.971	-2.885	5.198
90	Au	-0.758	2.334	6.715
91	Au	-1.517	4.668	5.198
92	Au	-0.758	-2.334	6.715
93	Au	-1.517	-4.668	5.198
94	Au	-2.454	0.000	6.715
95	Au	-4.908	0.000	5.198
96	Au	0.758	-2.334	-6.715
97	Au	1.517	-4.668	-5.198
98	Au	2.454	0.000	-6.715
99	Au	4.908	0.000	-5.198
100	Au	0.758	2.334	-6.715
101	Au	1.517	4.668	-5.198
102	Au	-1.985	1.442	-6.715
103	Au	-3.971	2.885	-5.198
104	Au	4.729	-5.219	1.227
105	Au	3.502	-6.110	-1.227
106	Au	6.425	-2.885	1.227
107	Au	6.894	-1.442	-1.227

108	Au	3.213	-5.219	3.681
109	Au	0.469	-6.110	3.681
110	Au	-3.213	5.219	-3.681
111	Au	-0.469	6.110	-3.681
112	Au	-4.729	5.219	-1.227
113	Au	-3.502	6.110	1.227
114	Au	-6.425	2.885	-1.227
115	Au	-6.894	1.442	1.227
116	Au	-0.758	7.002	1.227
117	Au	0.758	7.002	-1.227
118	Au	-3.971	4.668	3.681
119	Au	-5.667	2.334	3.681
120	Au	3.971	-4.668	-3.681
121	Au	5.667	-2.334	-3.681
122	Au	0.758	-7.002	-1.227
123	Au	-0.758	-7.002	1.227
124	Au	-3.971	-4.668	3.681
125	Au	-5.667	-2.334	3.681
126	Au	3.971	4.668	-3.681
127	Au	5.667	2.334	-3.681
128	Au	3.971	0.000	5.198
129	Au	6.425	0.000	1.227
130	Au	5.198	3.776	-1.227
131	Au	1.985	6.110	1.227
132	Au	1.227	3.776	5.198
133	Au	-1.985	-6.110	-1.227
134	Au	-1.227	-3.776	-5.198
135	Au	-3.971	0.000	-5.198
136	Au	-6.425	0.000	-1.227
137	Au	-5.198	-3.776	1.227
138	Au	1.227	-3.776	5.198
139	Au	-3.213	-2.334	5.198
140	Au	-3.213	2.334	5.198
141	Au	-1.227	3.776	-5.198
142	Au	3.213	2.334	-5.198
143	Au	3.213	-2.334	-5.198
144	Au	1.985	-6.110	1.227
145	Au	5.198	-3.776	-1.227
146	Au	-1.985	6.110	-1.227
147	Au	-5.198	3.776	1.227

Table S6. Optimized geometry of Pt_{147} structure with density functional theory at LDA/DZ level of theory.

	atom	x	y	z
1	Pt	0.000	0.000	0.000
2	Pt	1.985	1.442	1.227
3	Pt	0.000	0.000	2.744
4	Pt	1.985	-1.442	1.227
5	Pt	0.758	-2.334	-1.227
6	Pt	2.454	0.000	-1.227
7	Pt	0.758	2.334	-1.227
8	Pt	-0.758	2.334	1.227
9	Pt	-0.758	-2.334	1.227
10	Pt	0.000	0.000	-2.744
11	Pt	-2.454	0.000	1.227
12	Pt	-1.985	-1.442	-1.227
13	Pt	-1.985	1.442	-1.227
14	Pt	3.971	2.885	2.454
15	Pt	0.000	0.000	5.488
16	Pt	3.971	-2.885	2.454
17	Pt	1.517	-4.668	-2.454
18	Pt	4.908	0.000	-2.454
19	Pt	1.517	4.668	-2.454
20	Pt	-1.517	4.668	2.454
21	Pt	-1.517	-4.668	2.454
22	Pt	0.000	0.000	-5.488
23	Pt	-4.908	0.000	2.454
24	Pt	-3.971	-2.885	-2.454
25	Pt	-3.971	2.885	-2.454
26	Pt	1.985	1.442	3.971
27	Pt	3.971	0.000	2.454
28	Pt	4.440	1.442	0.000
29	Pt	2.744	3.777	0.000
30	Pt	1.227	3.777	2.454
31	Pt	-1.227	-3.777	-2.454
32	Pt	-2.744	-3.777	0.000
33	Pt	-1.985	-1.442	-3.971
34	Pt	-4.440	-1.442	0.000
35	Pt	-3.971	0.000	-2.454
36	Pt	1.985	-1.442	3.971
37	Pt	-0.758	2.334	3.971
38	Pt	-0.758	-2.334	3.971
39	Pt	-2.454	0.000	3.971
40	Pt	0.758	-2.334	-3.971
41	Pt	2.454	0.000	-3.971
42	Pt	0.758	2.334	-3.971
43	Pt	-1.985	1.442	-3.971
44	Pt	2.744	-3.777	0.000
45	Pt	4.440	-1.442	0.000
46	Pt	1.227	-3.777	2.454
47	Pt	-1.227	3.777	-2.454
48	Pt	-2.744	3.777	0.000
49	Pt	-4.440	1.442	0.000
50	Pt	0.000	4.668	0.000
51	Pt	-3.212	2.334	2.454
52	Pt	3.212	-2.334	-2.454

53	Pt	0.000	-4.668	0.000
54	Pt	-3.212	-2.334	2.454
55	Pt	3.212	2.334	-2.454
56	Pt	5.956	4.327	3.681
57	Pt	0.000	0.000	8.231
58	Pt	5.956	-4.327	3.681
59	Pt	2.275	-7.002	-3.681
60	Pt	7.362	0.000	-3.681
61	Pt	2.275	7.002	-3.681
62	Pt	-2.275	7.002	3.681
63	Pt	-2.275	-7.002	3.681
64	Pt	0.000	0.000	-8.231
65	Pt	-7.362	0.000	3.681
66	Pt	-5.956	-4.327	-3.681
67	Pt	-5.956	4.327	-3.681
68	Pt	3.971	2.885	5.198
69	Pt	1.985	1.442	6.715
70	Pt	5.956	1.442	3.681
71	Pt	5.956	-1.442	3.681
72	Pt	6.425	2.885	1.227
73	Pt	6.894	1.442	-1.227
74	Pt	4.729	5.219	1.227
75	Pt	3.502	6.111	-1.227
76	Pt	3.212	5.219	3.681
77	Pt	0.469	6.111	3.681
78	Pt	-3.212	-5.219	-3.681
79	Pt	-0.469	-6.111	-3.681
80	Pt	-4.729	-5.219	-1.227
81	Pt	-3.502	-6.111	1.227
82	Pt	-3.971	-2.885	-5.198
83	Pt	-1.985	-1.442	-6.715
84	Pt	-6.425	-2.885	-1.227
85	Pt	-6.894	-1.442	1.227
86	Pt	-5.956	-1.442	-3.681
87	Pt	-5.956	1.442	-3.681
88	Pt	1.985	-1.442	6.715
89	Pt	3.971	-2.885	5.198
90	Pt	-0.758	2.334	6.715
91	Pt	-1.517	4.668	5.198
92	Pt	-0.758	-2.334	6.715
93	Pt	-1.517	-4.668	5.198
94	Pt	-2.454	0.000	6.715
95	Pt	-4.908	0.000	5.198
96	Pt	0.758	-2.334	-6.715
97	Pt	1.517	-4.668	-5.198
98	Pt	2.454	0.000	-6.715
99	Pt	4.908	0.000	-5.198
100	Pt	0.758	2.334	-6.715
101	Pt	1.517	4.668	-5.198
102	Pt	-1.985	1.442	-6.715
103	Pt	-3.971	2.885	-5.198
104	Pt	4.729	-5.219	1.227
105	Pt	3.502	-6.111	-1.227
106	Pt	6.425	-2.885	1.227
107	Pt	6.894	-1.442	-1.227

108	Pt	3.212	-5.219	3.681
109	Pt	0.469	-6.111	3.681
110	Pt	-3.212	5.219	-3.681
111	Pt	-0.469	6.111	-3.681
112	Pt	-4.729	5.219	-1.227
113	Pt	-3.502	6.111	1.227
114	Pt	-6.425	2.885	-1.227
115	Pt	-6.894	1.442	1.227
116	Pt	-0.758	7.002	1.227
117	Pt	0.758	7.002	-1.227
118	Pt	-3.971	4.668	3.681
119	Pt	-5.667	2.334	3.681
120	Pt	3.971	-4.668	-3.681
121	Pt	5.667	-2.334	-3.681
122	Pt	0.758	-7.002	-1.227
123	Pt	-0.758	-7.002	1.227
124	Pt	-3.971	-4.668	3.681
125	Pt	-5.667	-2.334	3.681
126	Pt	3.971	4.668	-3.681
127	Pt	5.667	2.334	-3.681
128	Pt	3.971	0.000	5.198
129	Pt	6.425	0.000	1.227
130	Pt	5.198	3.777	-1.227
131	Pt	1.985	6.111	1.227
132	Pt	1.227	3.777	5.198
133	Pt	-1.985	-6.111	-1.227
134	Pt	-1.227	-3.777	-5.198
135	Pt	-3.971	0.000	-5.198
136	Pt	-6.425	0.000	-1.227
137	Pt	-5.198	-3.777	1.227
138	Pt	1.227	-3.777	5.198
139	Pt	-3.212	-2.334	5.198
140	Pt	-3.212	2.334	5.198
141	Pt	-1.227	3.777	-5.198
142	Pt	3.212	2.334	-5.198
143	Pt	3.212	-2.334	-5.198
144	Pt	1.985	-6.111	1.227
145	Pt	5.198	-3.777	-1.227
146	Pt	-1.985	6.111	-1.227
147	Pt	-5.198	3.777	1.227

Table S7. The Lennard-Jones parameters for nanoparticles simulation.

atom	ε(kJ mol^{-1})	σ(nm)
Ag[1]	19.05865	0.2995
Au[2]	22.13300	0.2629
Pt[3]	19.42249	0.2410

Table S8. The interaction energy of HCQ and CQ molecules with 2-layer slab model of Ag (111), Au (111), and Pt (111).

Structure	ΔE_b (eV)
Ag(111) - CQ	-1.43
Ag(111) - HCQ	-1.75
Au(111) - CQ	-2.45
Au(111) - HCQ	-2.38
Pt(111) - CQ	-4.15
Pt(111) - HCQ	-4.19

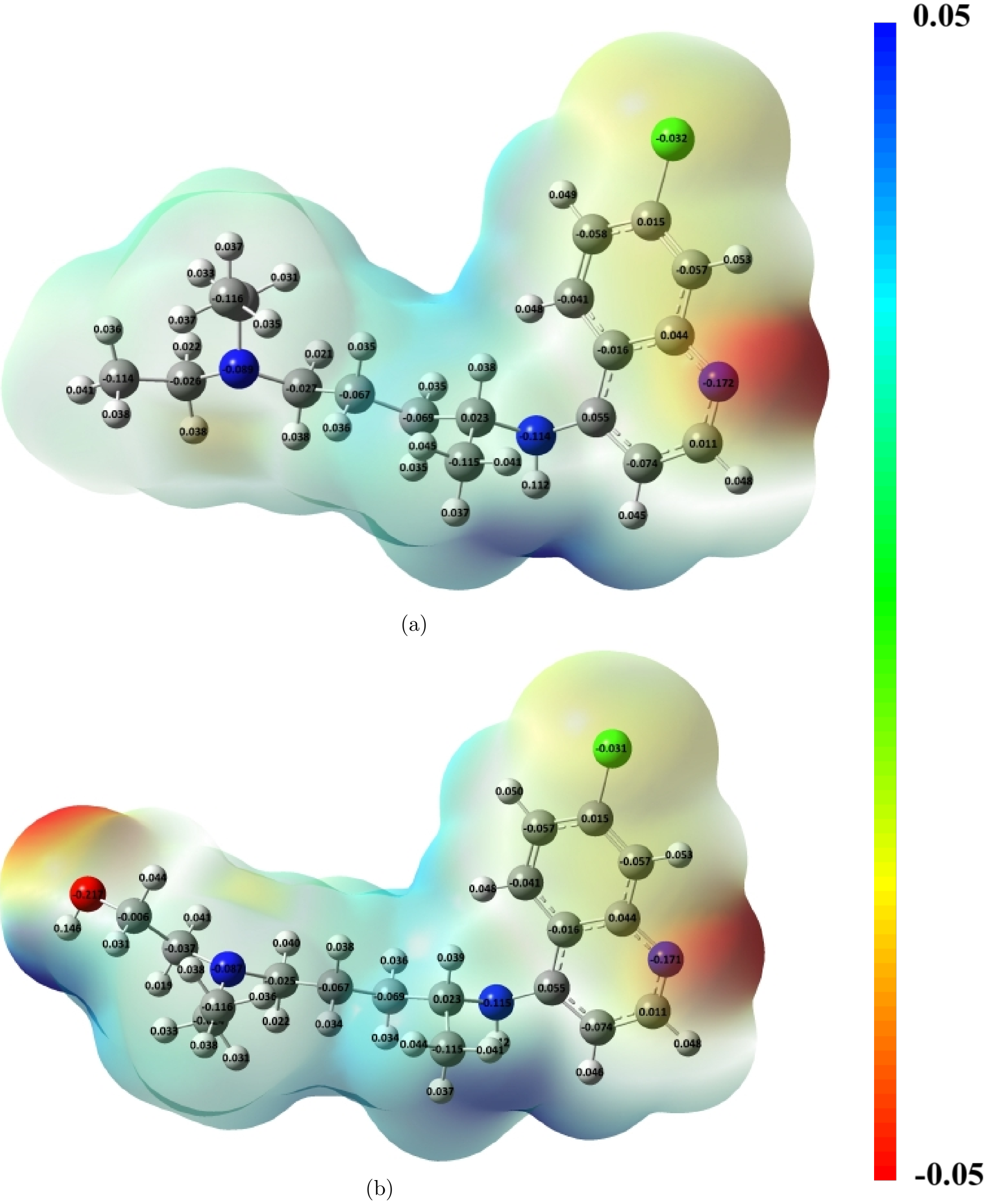

Fig. S1. Charge distribution of HCQ and CQ molecules and their electrostatic potential map (Iso–value = 0.0004 $e\text{Å}^{-3}$).

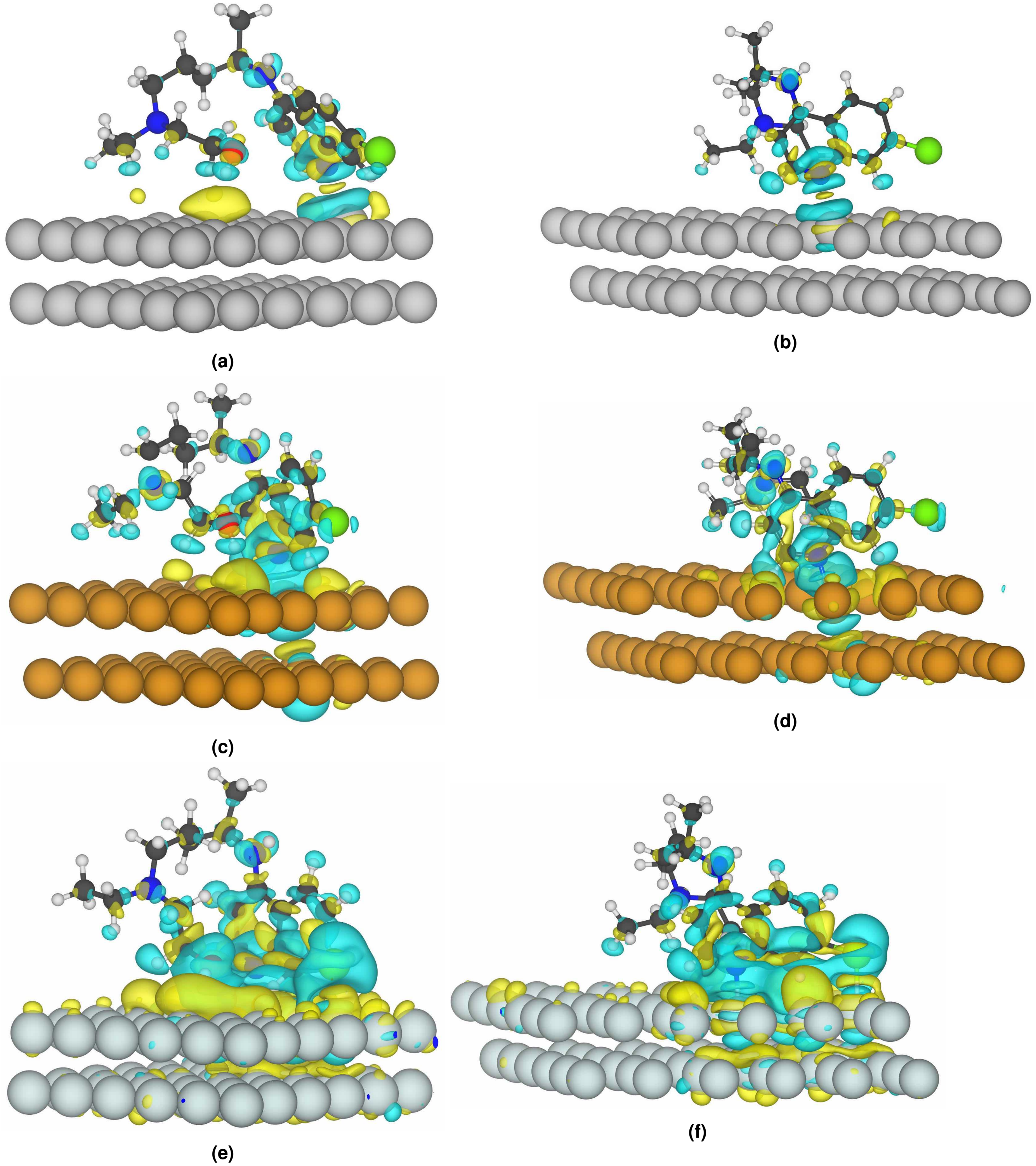

Fig. S2. The charge density difference of Adsorbed of HCQ /CQ molecules on a periodic slab model of the Ag(111), Au(111), and Pt(111). The isovalue for the charge transfer plot, is fixed at $0.001e/a.u^3$. Yellow and blue color indicate positive and negative level corresponds to gain and loss of electron density.

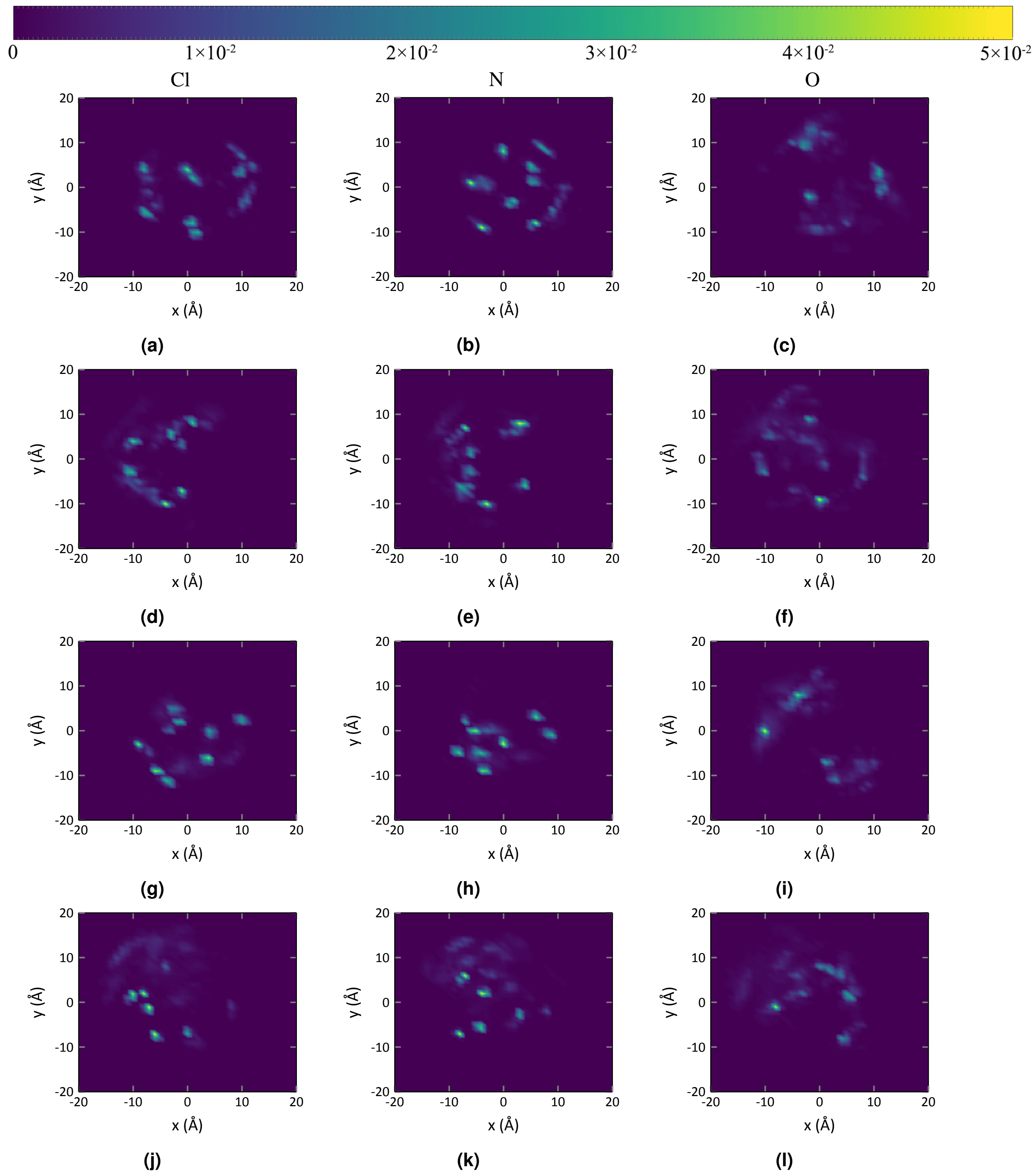

Fig. S3. Probability distribution of Cl, N (in pyridine ring) and O of HCQ near the (a–c) AgNP, (d–f) AuNP, (g–i) AuAgNP, and (j–l) PtNP.

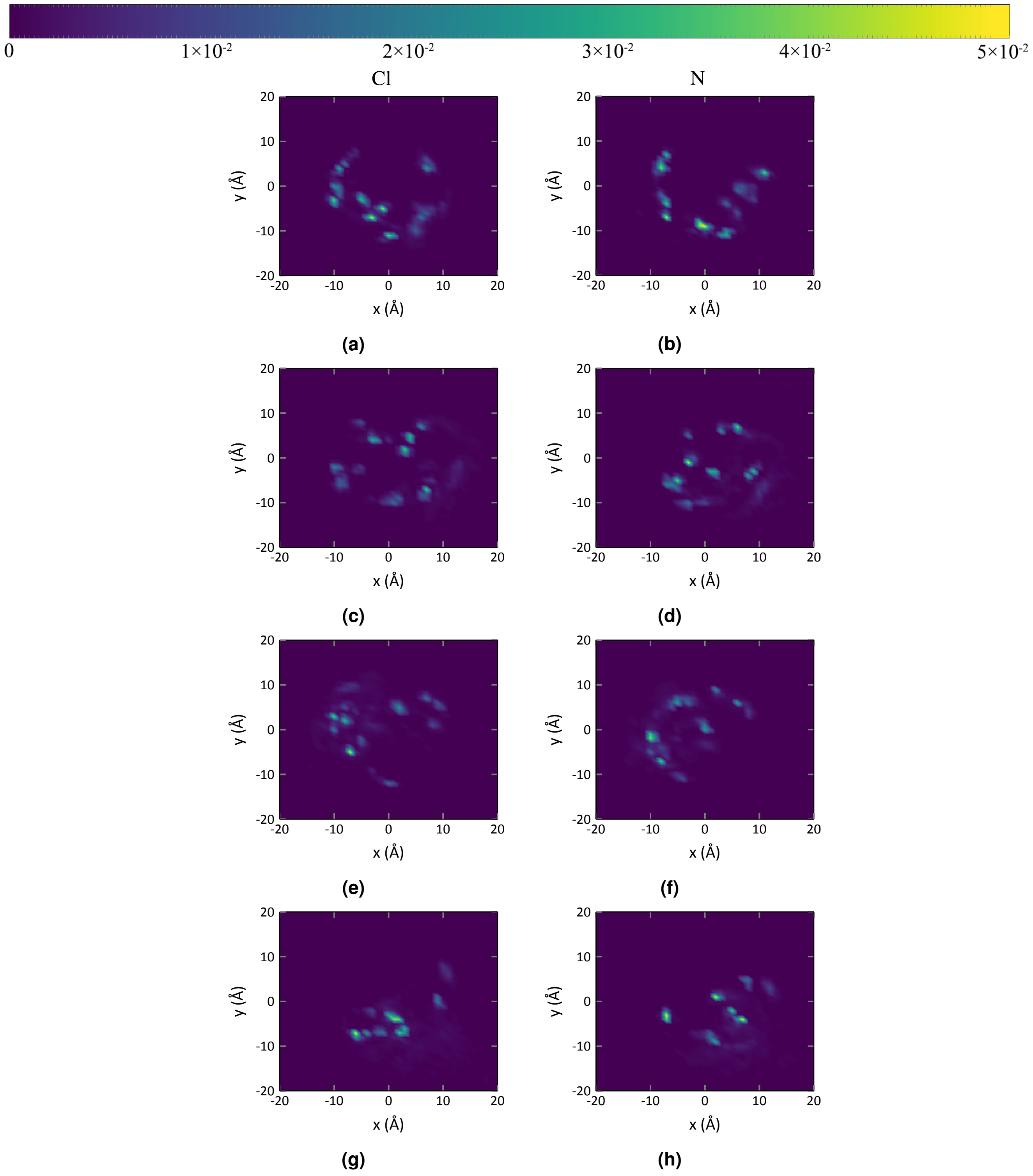

Fig. S4. Probability distribution of Cl and N (in pyridine ring) of CQ near the (a,b) AgNP, (c,d) AuNP, (e,f) AuAgNP, and (g,h) PtNP.

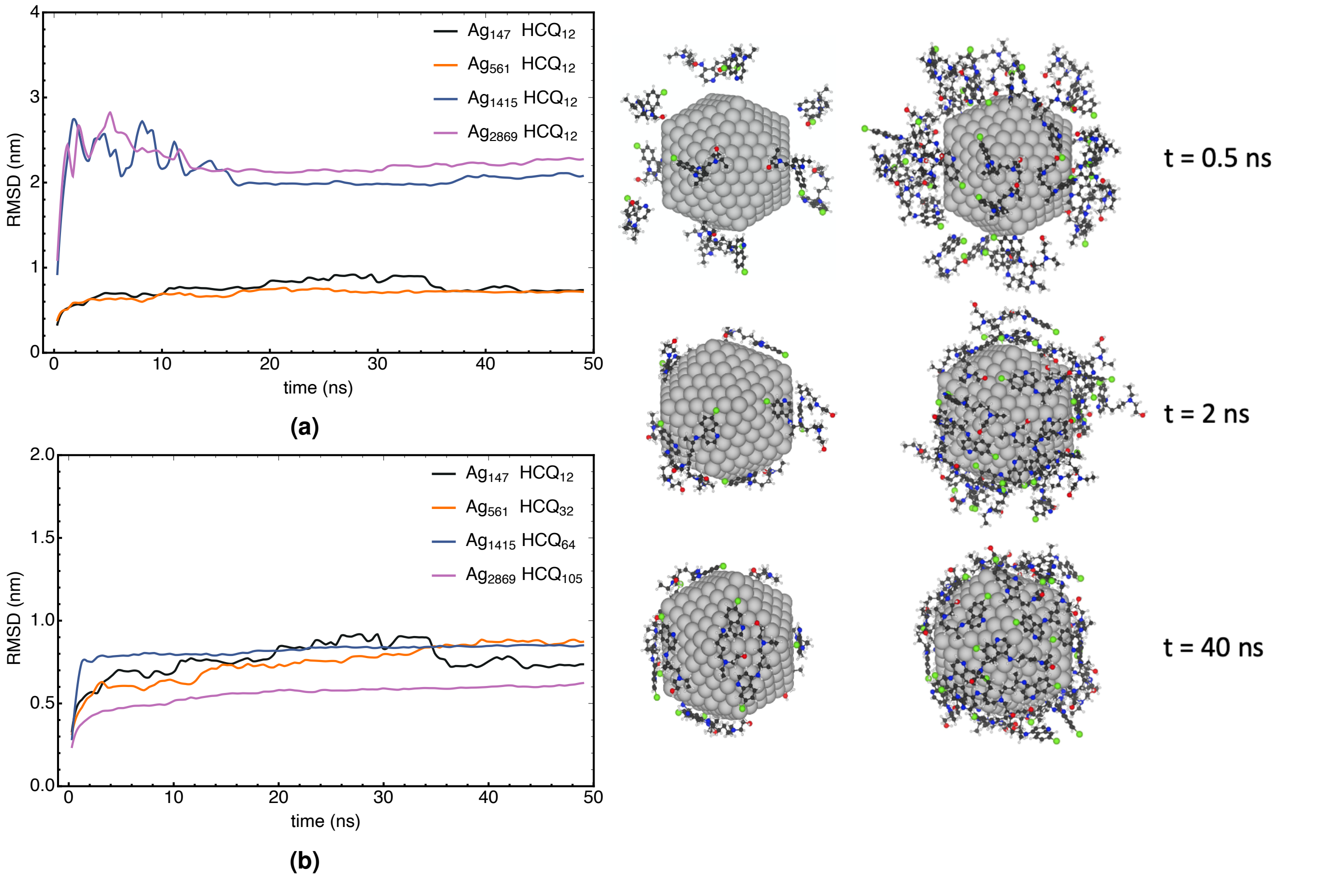

Fig. S5. RMSD of different size of AgNPs coated with HCQs. (a) fixed number of HCQs (12) (b) increasing number of HCQ proportional to the Ag atom on the surface of NPs.

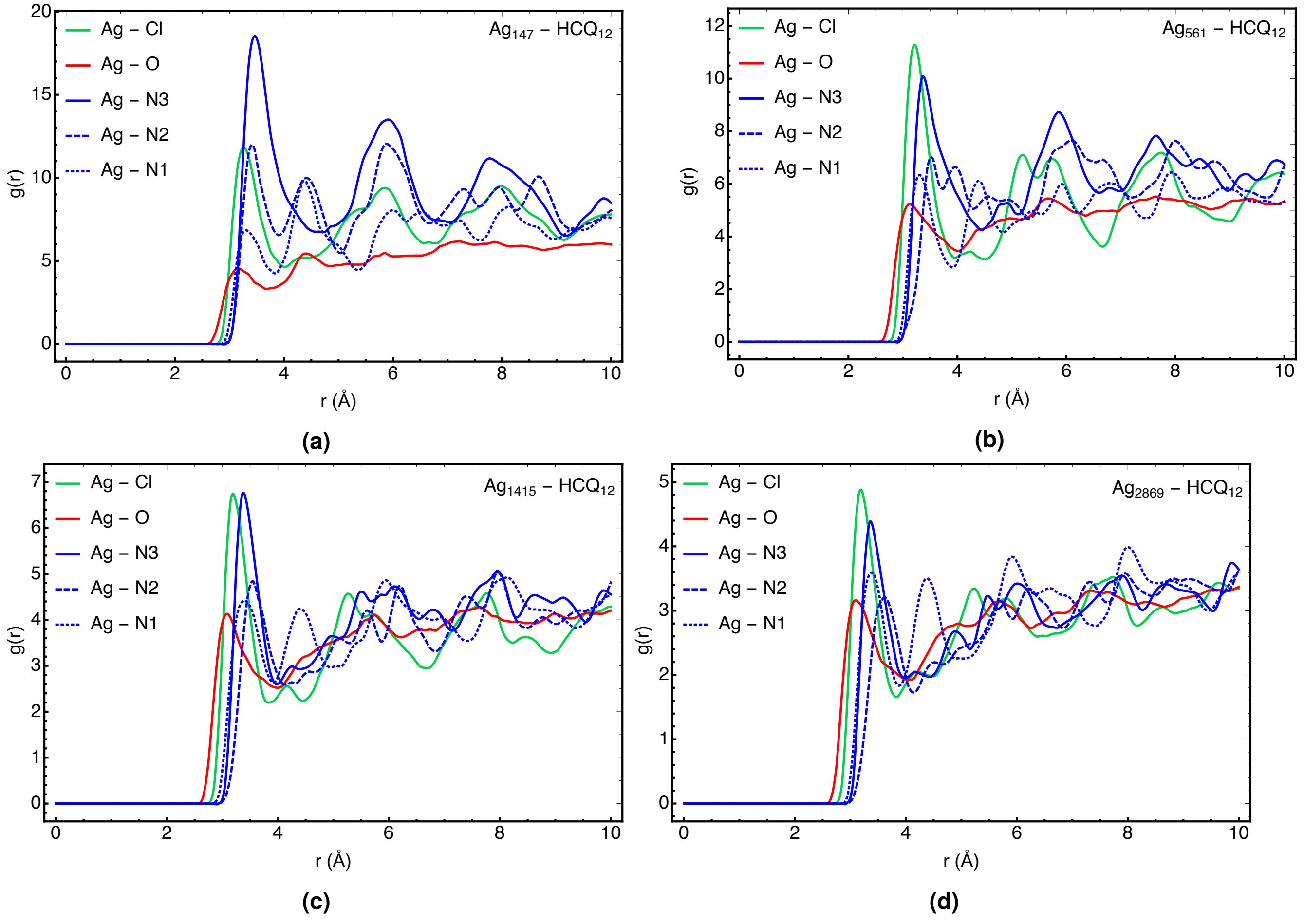

Fig. S6. The comparison of RDFs for (a) Ag_{147}, (b) Ag_{561}, (c) Ag_{1415} and (d) Ag_{2869} with 12 molecules of HCQ.

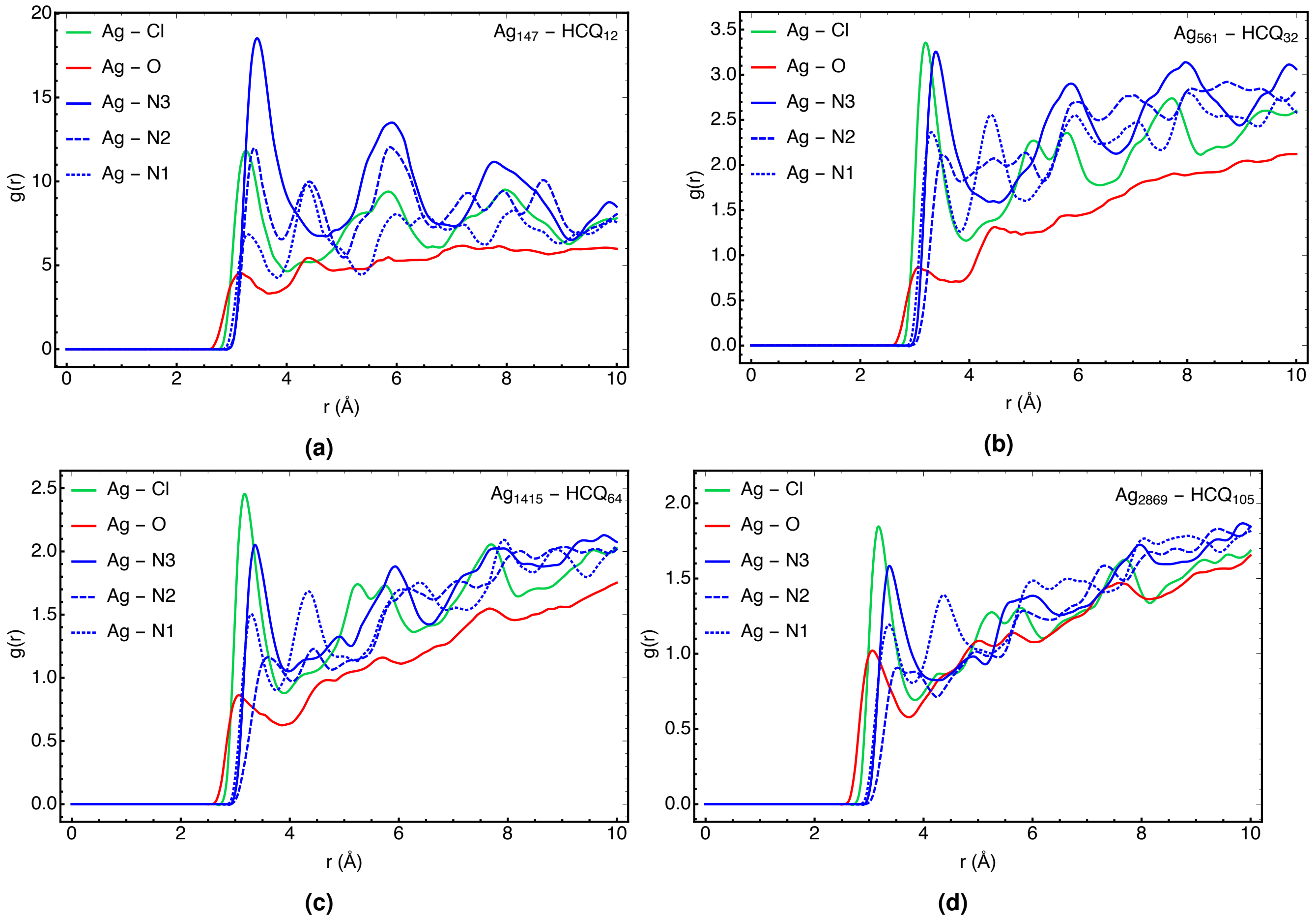

Fig. S7. The comparison of RDFs for (a) Ag_{147}, (b) Ag_{561}, (c) Ag_{1415} and (d) Ag_{2869} with 12, 32, 64 and 105 molecules of HCQ.

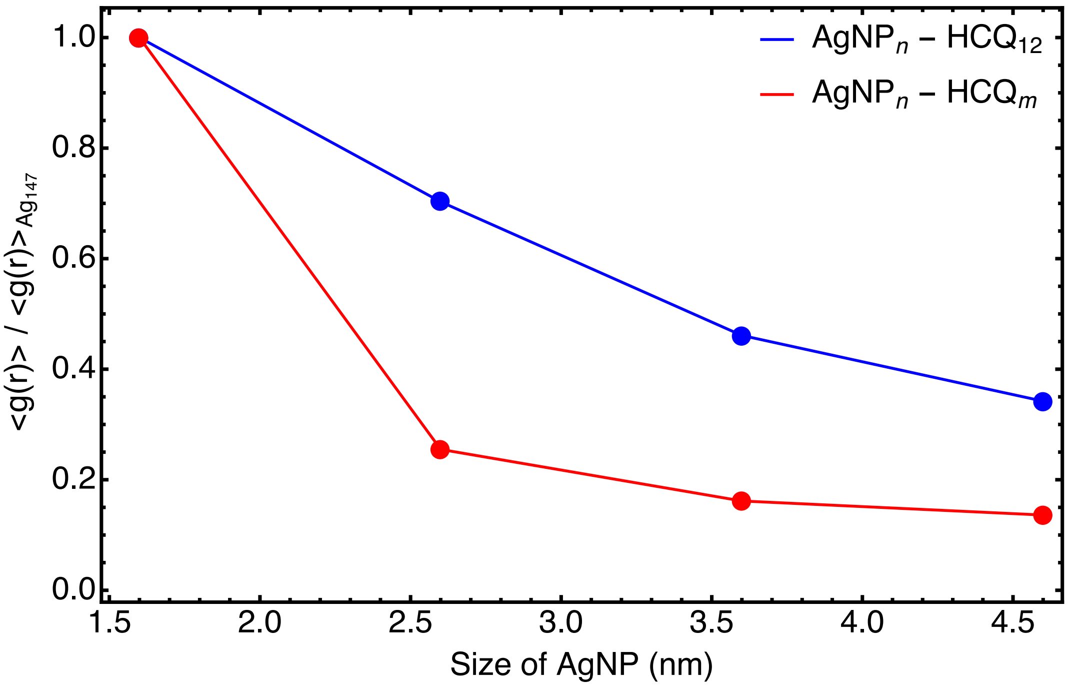

Fig. S8. Decreasing the overall coating properties by increasing the size of AgNP from 1.6 (Ag_{147}) to 4.6 (Ag_{2869}) nm. The mean values of RDF are calculated using Eq. S1 and S2.

$$\bar{R}_n = \frac{\int_0^{10} dr\, g(r)\,|_{Ag_n - HCQ_{12}}}{\int_0^{10} dr\, g(r)\,|_{Ag_{147} - HCQ_{12}}}\,. \tag{S1}$$

$$\bar{R}_{n,m} = \frac{\int_0^{10} dr\, g(r)\,|_{Ag_n - HCQ_m}}{\int_0^{10} dr\, g(r)\,|_{Ag_{147} - HCQ_{12}}}\,. \tag{S2}$$

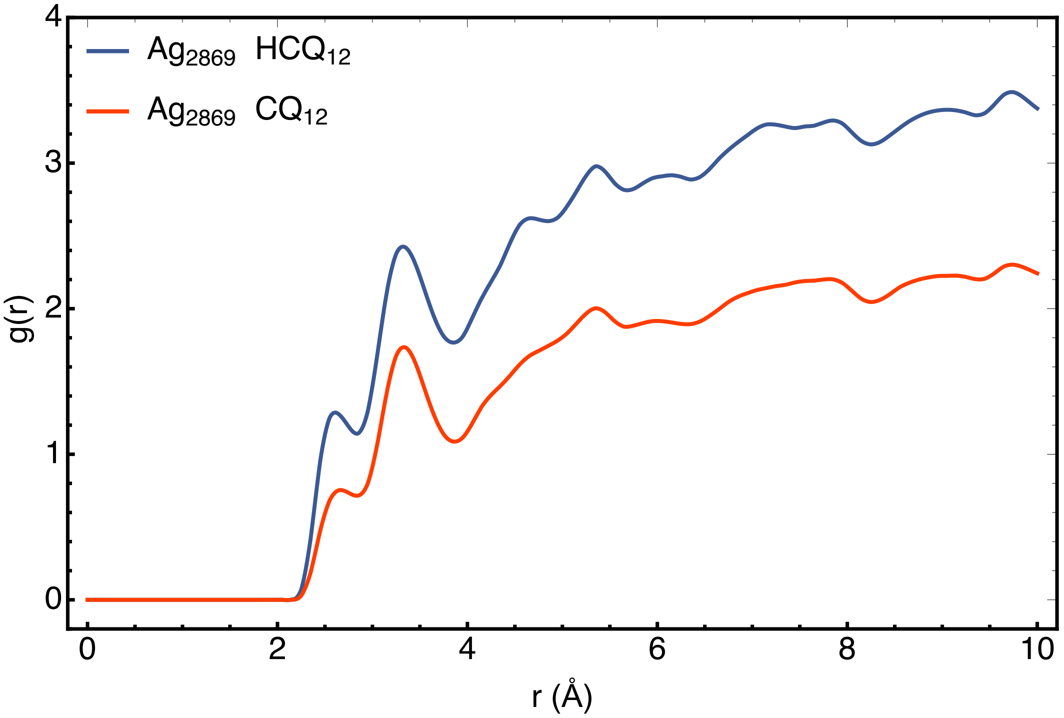

Fig. S9. The comparison of RDFs for HCQ and CQ with Ag_{2869} .

References

1. Kyrychenko, A., Pasko, D. A. & Kalugin, O. N. Poly(vinyl alcohol) as a water protecting agent for silver nanoparticles: the role of polymer size and structure. *Phys. Chem. Chem. Phys.* **19**, 8742–8756, DOI: 10.1039/C6CP05562A (2017).

2. Phanchai, W., Srikulwong, U., Chompoosor, A., Sakonsinsiri, C. & Puangmali, T. Insight into the Molecular Mechanisms of AuNP-Based Aptasensor for Colorimetric Detection: A Molecular Dynamics Approach. *Langmuir* **34**, 6161–6169, DOI: 10.1021/acs.langmuir.8b00701 (2018).

3. Callejas-Tovar, R., Liao, W., Martinez de la Hoz, J. M. & Balbuena, P. B. Molecular Dynamics Simulations of Surface Oxidation on Pt(111) and Pt/PtCo/Pt $_3$ Co(111). *The J. Phys. Chem. C* **115**, 4104–4113, DOI: 10.1021/jp110436e (2011).
